# Beyond the Average: Modeling Individual-Specific Preferences for Ulcerative Colitis Surgery

**DOI:** 10.1016/j.jval.2026.02.010

**Published:** 2026-08

**Authors:** Nyantara Wickramasekera, Donna Rowen, Steven Brown, Arne Risa Hole

**Affiliations:** 1 Sheffield Center for Health and Related Research, University of Sheffield, Sheffield, England, UK; 2 Sheffield Teaching Hospitals NHS Foundation Trust, Sheffield, England, UK; 3 School of Economics, Jaume I University, Spain

**Keywords:** conditional probability, preference diagnostic, preference heterogeneity

## Abstract

**Objectives:**

Surgical decisions for ulcerative colitis are complex and preference-sensitive. This study aimed to assess patient preferences for surgical treatments, quantify preference heterogeneity, and examine individual-specific preferences to inform decision making.

**Methods:**

Patient preferences were elicited using a discrete choice experiment. A rigorous selection process involving focus groups and interviews with clinicians and patients resulted in 7 key attributes. Each task included 2 unlabeled surgical alternatives and a medication opt-out. A D-efficient fractional factorial design was generated. The survey was pilot tested using “think-aloud” interviews. Data were analyzed using multinomial and mixed logit models, with conditional mean coefficients used to estimate individual-specific choice probabilities.

**Results:**

Three hundred and fifty patients completed the survey. Results showed significant preference heterogeneity for most attributes. The preference for the medication opt-out revealed a multimodal conditional distribution, clustering patients who strongly preferred, were indifferent to, or disliked medication. The interaction term “planning to have children” fully explained the preference heterogeneity in the fertility attribute. A gender interaction term showed that male patients had a stronger negative preference for a stoma. Choice probabilities showed individual differences; some patients had a 97.98% probability of preferring medication, whereas others had only a 0.09% probability, instead showing a high preference for surgical options.

**Conclusions:**

This study demonstrates the value of using conditional distributions to examine preference heterogeneity. Simpler models failed to reveal the wide range of preferences present in the data. Conditional choice probabilities can be used to better understand how different patients make treatment decisions.

## Highlights

• Mixed logit models are commonly used in health preference elicitation studies. However, the quantification of heterogeneity is typically limited to the means and standard deviations of the coefficient distributions.
• This study uses mixed logit models to reveal preference heterogeneity for surgical treatments for ulcerative colitis using conditional mean coefficients. Individual-specific choice probabilities show distinct preference patterns: some patients have a high probability of preferring medication, whereas others have a near-zero probability of choosing medication, indicating a strong preference for surgery. In the overall population, subtotal colectomy was preferred, followed by ileorectal anastomosis.
• Moving beyond mean parameter estimates provides nuanced insights into individual-specific preferences for surgical treatments. This method offers clinical applications, particularly through its integration into decision-support tools.

## Introduction

Ulcerative colitis (UC) is a chronic inflammatory bowel disease, characterized by cycles of active symptoms followed by periods of remission.^1^ Although many patients manage their UC with medication, approximately 20% to 30% of patients will require surgical intervention, in which all or part of their bowel is removed.^1^^,^^2^ When medical management fails, patients face a complex and preference-sensitive decision involving trade-offs among various surgical options. These include the option of having reconstructive surgery, such as ileal pouch-anal anastomosis, ileorectal anastomosis, or procedures resulting in a stoma, such as subtotal colectomy or proctocolectomy.^1^

Discrete choice experiments (DCEs), a stated preference method, are widely used in the health literature to elicit preferences for treatments.^3^ A key advantage of DCEs over other methods, such as time trade-off, is the ability to model data that account for both observed and unobserved heterogeneity across multiple attributes simultaneously.^4^ This allows for an exploration of preferences beyond the population average, enabling the estimation of individual or group-level preferences to better understand how different patients make treatment decisions.^5^

The surgical decision for UC is preference-sensitive because no single procedure is universally superior for all patients.^2^ The choice depends on a patient’s values and treatment history, requiring them to weigh competing treatment factors, such as symptom resolution against drawbacks such as loss of natural bowel function, lifestyle adjustments, and complication risks. Considerable variability exists in how patients trade off these potential advantages and disadvantages.^6^, ^7^, ^8^, ^9^ For example, some patients may have an overwhelming aversion to a permanent stoma, whereas others may prioritize minimizing bowel frequency. Because some of this preference heterogeneity may be unrelated to observable personal characteristics, ignoring this individual variability can bias model estimates.

Only a limited number of studies have explored quantitative patient preferences for surgical treatment options in UC.^7^^,^^10^ This contrasts with the majority of previous patient preference studies, which have predominantly focused on preferences comparing medical treatments for UC.^10^^,^^11^ One DCE study found that patients with UC are willing to trade off increased mortality risks from medical treatment to avoid a permanent stoma, and found the outcome of a reconstructive surgery to be equivalent to living with mild disease activity managed by medication.^12^ Another study (treatment trade-off threshold) showed that patients are willing to accept increased surgical complication risks, such as anastomotic leak or pouch failure, to avoid a temporary diverting ileostomy for ileal pouch-anal anastomosis surgery.^13^ Although sociodemographic factors and treatment history explain some variation in patient preferences, individuals likely possess different underlying preferences, with much of this heterogeneity stemming from unobservable characteristics. This heterogeneity is largely unexamined in the existing surgical literature. To address this gap, the primary aim of this study is to assess patient preferences for surgical treatments and their underlying preference heterogeneity. The secondary aim is to examine individual-specific preferences, providing a more nuanced understanding of individual patient decision making.

## Methods

Patient preferences were elicited using the DCE method. Respondents are presented with 2 or more alternatives, each described by its key characteristics (attributes) in a side-by-side comparison table.^3^^,^^14^^,^^15^ Patients are then asked to weigh these alternatives and select the alternative that they prefer. These choice data are econometrically modeled to understand the relative importance of the attributes and the trade-offs respondents are willing to make. This study followed established principles of good practice for DCEs recommended by the ISPOR Task Force^16^^,^^17^ and is reported in accordance with the DIRECT^18^ checklist (see Appendix Table 1 in Supplementary Materials).

### Attribute Selection

A DCE can meaningfully include only a limited number of important attributes.^15^ A rigorous selection process was used to reduce the list of attributes and select the most appropriate attributes for the DCE. This process involved focus groups, individual interviews, and meetings with clinicians and patients. Senior clinicians working in both surgery and medical teams provided input. The patient involvement group consisted of patients either taking medication or who had surgery for UC. To ensure diversity of views, patients were recruited from different genders, age groups, and ethnic minority backgrounds. Initially, a literature review yielded 20 potential attributes (see Appendix Table 2 in Supplementary Materials). These were subsequently reduced using a combination of a modified nominal group technique^20^ and individual ranking of attributes. First, in the “information giving” stage, literature review findings were presented to 10 patients and 4 clinicians. Second, in the “share idea” stage, views were exchanged about what attributes were more important and less important. In the final “prioritization” stage, patients and clinicians ranked or expressed their preferences for what attributes should be included in the final DCE. Through this iterative process, less important attributes were removed, and similar attributes were merged. For example, the list of surgical complications was consolidated into short-term acute events (defined by Clavien-Dindo grades III-IV) and long-term complications, such as treatment failures occurring after the 30-day perioperative period, as recommended by clinicians.^21^ Further details about the selection process are included in Appendix Table 2 in Supplementary Materials. After this iterative process, 7 attributes were included in the DCE (Appendix Table 3 in Supplementary Materials). Attribute levels were identified from the literature^22^, ^23^, ^24^, ^25^, ^26^ and refined through consultations with clinical experts.

### DCE Design and Survey

**Figure 1:**
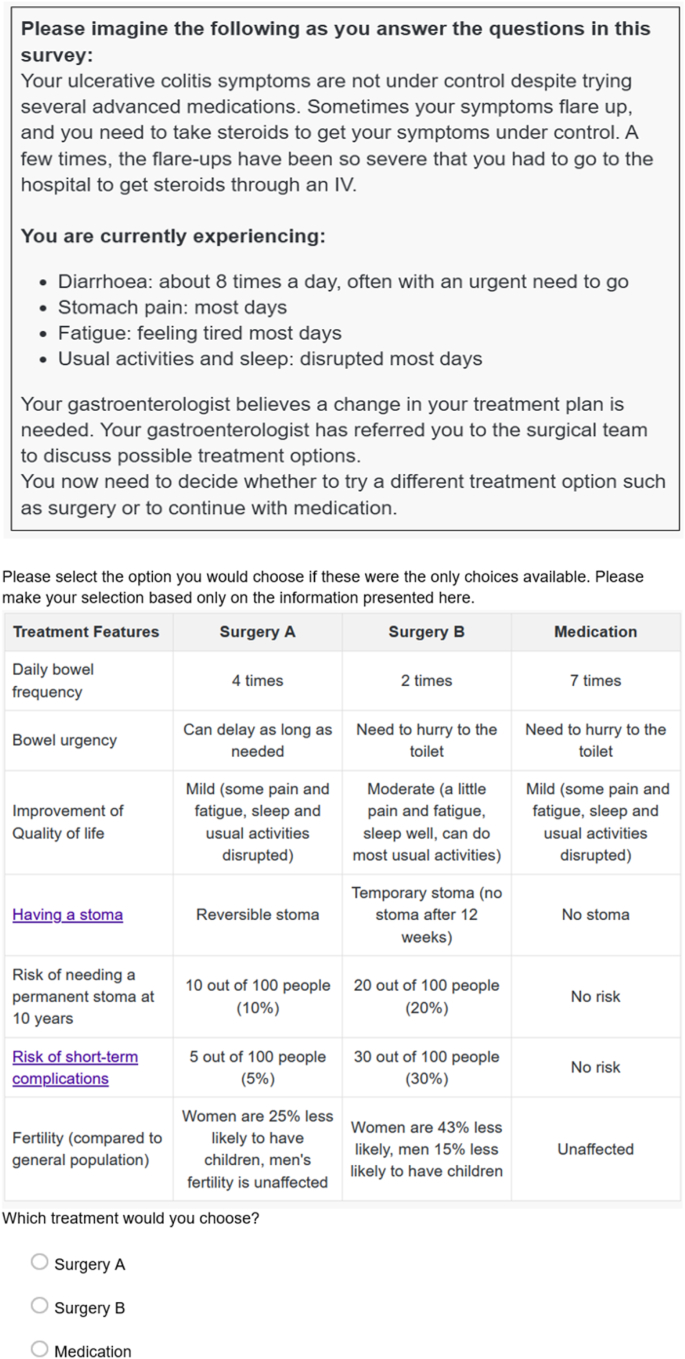
DCE scenario and task.

The attribute levels for each DCE task were generated using the Ngene software.^27^ A D-efficient, main effects, fractional factorial design using directional priors was created.^28^ Each task included 2 unlabeled surgical alternatives, with a medical alternative opt-out providing the reference level (Fig. 1). The medical opt-out was included to mimic the actual decision-making context a patient would face. In a real-world setting, patients facing persistent symptoms despite multiple medications (Fig. 1) have the choice between a surgical procedure or continuing with medication. A design containing 36 total tasks, blocked into 4 sets of 9 tasks, was selected based on low D-error scores. Detailed information on the DCE survey sections can be found in Appendices 4 and 5 in Supplementary Materials.

### Pilot

The Qualtrics survey was pilot tested with 8 members of the patient involvement group who had not participated in the attribute selection process. These 1-to-1 “think-aloud” interviews aimed to evaluate comprehension of the DCE task, assess the task burden, and ensure clarity in the framing of the DCE questions by asking respondents to verbalize their thought processes as they complete the survey.^29^ Patient feedback was subsequently incorporated to improve the acceptability, usability, and comprehensibility of the DCE survey before proceeding to the data collection stage.

Several modifications were implemented during the piloting phase. First, the opt-out medication alternative was amended to include the reference level for each attribute, addressing patient concerns that the initially blank column made the opt-out look incomplete (see Appendix 6 in Supplementary Materials). Second, to mitigate participant burden associated with the complex decision context, the number of choice tasks per survey was decreased from 12 to 9. Third, the wording of survey instructions was simplified to enhance comprehension, with a focus on using simple language and concise sentence structure. After this, the survey achieved a Flesch Reading Ease score of 59, indicating readability for individuals aged 15 and above.^30^^,^^31^ Fourth, explanations were added to clarify the meaning of “mild,” “moderate,” and “high” quality of life levels within the DCE table. To minimize ambiguity, the quality of life attribute was defined by symptoms such as pain, fatigue, and sleep, clearly separating it from the other efficacy attributes of bowel urgency and frequency. Finally, hyperlinks were added to the DCE table to provide more detailed attribute information that participants could refer to throughout the DCE tasks.

### DCE Data Collection

Potential participants were recruited from the National Institute for Health and Care Research Be Part of Research registry, a database of UC patients expressing interest in taking part in research. An invitation email, containing a link to the Qualtrics survey, was sent to 2409 individuals. Eligibility criteria included UK residency, age 16 or above, and self-reported UC diagnosis. Including all UC patients reflects the relapsing and remitting nature of the disease because severe worsening of the disease makes surgery a potential treatment consideration. Although official diagnosis verification was not performed, no monetary incentives were offered for taking part in the study, either by the study team or the Be Part of Research registry. A target sample size of around 300 was selected, aligning with literature indicating that most DCE studies utilize sample sizes between 100-300.^32^ Quotas were introduced to ensure a balance of males and females in the sample.

### Data Analysis

The relationship between patient choices and treatment attributes from the DCE was analyzed using multinomial logit, mixed logit, and latent class models (see Appendix 7 in Supplementary Materials). Several model specifications were evaluated (see Appendix 8 in Supplementary Materials), with the final model incorporating 4 dummy-coded categorical attributes and 3 linear attributes. Preference heterogeneity was examined in relation to both observed factors (through sociodemographic interactions) and unobserved factors (using continuous random heterogeneity). Mixed logit models were estimated by changing distributional assumptions (normal, log-normal distributions) and by changing the number of draws (see Appendix 9 in Supplementary Materials). The mixed logit model results were further analyzed to show the conditional coefficient distributions and conditional probabilities based on each individual’s observed choices. Data analyses were conducted using R (Apollo)^33^ and Stata (mixlogit) software.^34^ Summary statistics are presented for sociodemographic, quality assurance, and survey feedback variables.

Multinomial logit model:

(1) $$U_{njt}=asc_{njt}+{\beta_{1}B}_{njt}+{\beta_{2}I}_{njt}+{\beta_{3}Q}_{njt}+{\beta_{4}S}_{njt}+{\beta_{5}S}_{njt}M_{n}+{\beta_{6}L}_{njt}+{\beta_{7}R}_{njt}+{{{\beta_{8}F}_{njt}+\beta }_{9}F}_{njt}C_{n}+\varepsilon_{njt}$$

in which $U_{njt}$ is the utility that individual n obtains from choosing alternative j in choice situation t. The surgical attributes included in the observed part of the utility function are labelled B(bowelfrequency),I(urgency),Q(QoL),S(stoma),L(longrisk),R(shortrisk),F(fertility) (see Appendix Table 3 in Supplementary Materials for further details of the attributes) with corresponding coefficients $\beta_{1-9}$ estimating their relative importance. Individual characteristics M being male and C planning to have children are interacted with the attributes S(stoma),F(fertility), respectively. The alternative specific constant $acs_{nj}$ captures the preferences for the medication opt-out alternative. The error term $\varepsilon_{njt}$ captures the factors not included in the utility function and is assumed to be distributed iid extreme value.

Mixed logit model:

(2) $$U_{njt}=\beta_{n}^{\prime }x_{njt}+\varepsilon_{njt}$$

in which $U_{njt}$ is the utility that individual n gets from choosing alternative j in choice situation t. Although $X_{njt}$ are the observed variables as defined in equation 1 above, and $\beta_{n}$ is a vector of coefficients for the observed variables for individual n. The main difference in equation 2 is that the coefficients $\beta_{n}$ vary across individuals in the population, unlike the fixed coefficients of a multinomial logit model. This variation is represented by a density function g(β|θ) which describes the distribution of $\beta_{n}$ across the population. The analyst can specify the distributions of g(.) and can then estimate the parameters θ, this gives the parameters of the unconditional coefficient distributions for the population. The error term follows the same distribution as in Eq (1).

In addition to the population coefficient distributions, we can also estimate distributions of subpopulations or distributions of individual respondents, following Train.^5^ These distributions of coefficients for a group or an individual are called conditional distributions and are denoted by h(β|i,x,θ), in which β is the coefficient vector in the subpopulation of people who would choose alternative i when facing a choice situation described by x. To estimate the likely location of an individual on the unconditional distribution, we can use the sequence of observed choices for a given individual to calculate their posterior conditional distributions using Bayes’ rule, this can be denoted as:

(3) $$h\left(\beta |Y_{n},X_{n},\theta \right)=\frac{P\left(Y_{n}|X_{n},\beta \right)g\left(\beta |\theta \right)}{P\left(Y_{n}|X_{n},\theta \right)}$$

in which $Y_{n}$ represents the sequence of observed choices for a given individual, $X_{n}$ is a set of alternatives that correspond to those choices, and g(β|θ) is the unconditional distribution. From the conditional distribution, we can then calculate parameters such as the conditional mean coefficients. The mean vector of $h\left(\beta |Y_{n},X_{n},\theta \right)$ is:

(4) $${\bar{\beta }}_{n}=\int \beta h\left(\beta |Y_{n},X_{n},\theta \right)d\beta$$

in which the conditional mean vector is equal to the integral of beta, multiplied by the conditional density. Because this integral lacks an analytical solution, the conditional mean vector is estimated through simulation.

These conditional mean coefficients (from Eq. [4]) can then be used to calculate conditional probabilities. Following Train, section 11.6.3,^5^ McFadden’s logit formula^35^

(5) $$\frac{e^{\beta_{n}^{\prime }x_{nit}}}{\sum e^{\beta_{n}^{\prime }x_{njt}}}$$

was applied with the conditional mean coefficients.

## Results

A total of 350 patients completed the survey. This represents a 74% response rate (26% dropout rate) among those who clicked the Qualtrics link (350/472) and a 15% rate relative to all email invitations sent (350/2409). Table 1 describes the sample characteristics. The number of male and female respondents was roughly equal (47.1% male, 52.3% female), with a good distribution of responses from all age groups. The majority of participants were White (95.7%), married/partnered (66.0%), and employed (61.1%), with 46.0% educated with a bachelor’s degree or higher. About a third of the sample reported that they are planning to have children in the future. Patient treatment details showed that half of the sample were diagnosed with UC more than 10 years ago. The majority (91.7%) had not undergone surgery and were roughly equally divided between those in active disease (51.7%) and those in remission (48.3%).

**Table 1:** Characteristics of the sample.

Characteristics	No.	%
Gender		
Male	165	47.1%
Female	183	52.3%
Non-binary	2	0.6%
Age group		
16-24 years old	19	5.4%
25-34 years old	48	13.7%
35-44 years old	79	22.6%
45-54 years old	78	22.3%
55-64 years old	62	17.7%
65+ years old	64	18.3%
Employment		
In employment or self-employment	214	61.1%
Retired	76	21.7%
Looking after the home and family	9	2.6%
Taking care of a family member with chronic illness or disability	5	1.4%
Student	9	2.6%
Job-seeking (unemployed and looking for employment)	5	1.4%
Long-term sick or disabled	32	9.1%
Ethnicity		
White	335	95.7%
Black/African/Caribbean/Black British	1	0.3%
Asian/Asian British	7	2.0%
Mixed/Multiple ethnic groups	7	2.0%
Education		
Primary	3	0.9%
Secondary (GCSE/ O-Level)	66	18.9%
Further education (A-Level, BTEC)	118	33.7%
Bachelor’s, Master’s, Doctoral degree	161	46.0%
Prefer not to say	2	0.6%
Marital status		
Married/Partner	231	66.0%
Widowed	12	3.4%
Divorced/Separated	28	8.0%
Single	77	22.0%
Prefer not to say	2	0.6%
Plan to have children		
Yes	94	26.9%
No	240	68.6%
Prefer not to say	16	4.6%
How long have you been diagnosed with ulcerative colitis		
Less than 1 year	11	3.1%
1 to 2 years	28	8.0%
3 to 5 years	76	21.7%
6 to 10 years	56	16.0%
More than 10 years	179	51.1%
Have you had surgery to treat your ulcerative colitis		
Yes	29	8.3%
No	321	91.7%
Patients are in quiescent IBD (score of ≥13 for IBD-Control-8)^19^		
No	181	51.7%
Yes	169	48.3%

### Modeled Preferences

The results of the multinomial logit model and the mixed logit models are presented in Table 2. In general, the direction of the estimated preferences was as expected, where patients prefer a high improvement in quality of life after surgery and prefer procedures with better bowel control. Conversely, attributes such as higher levels of daily bowel frequency, a higher risk of needing a permanent stoma at 10 years, and a higher risk of short-term complications significantly decrease the likelihood of choosing surgery. Compared with not having a stoma, a permanent stoma has a large negative impact on the likelihood of choosing surgery, followed by a reversible stoma, and a temporary stoma. Although the multinomial logit model indicated a positive preference for the medication opt-out alternative, the mixed logit model revealed a significant negative preference on average. This divergence is attributable to the mixed logit model’s ability to capture unobserved preference heterogeneity. The large and significant standard deviation estimated for the opt-out parameter suggests that some people strongly preferred medication, whereas others did not. Moreover, significant preference heterogeneity was observed in all attributes except for the fertility attribute. This is because including the interaction term “planning to have children” accounted for all the preference heterogeneity in this attribute, resulting in mean and standard deviation parameters that are not statistically significant. In addition, the interaction term for gender shows that male patients have an even stronger negative preference for reversible stomas and permanent stomas compared with female/nonbinary respondents. The Akaike information criterion and Bayesian information criterion values indicate that the mixed logit models provide a better fit to the data compared with the multinomial logit model with interactions. The mixed logit model with log-normal distributions had the lowest Akaike information criterion and Bayesian information criterion values. Internal validity checks confirmed high data quality, with over 90% of respondents passing the quality assurance questions (see Appendix 10 in Supplementary Materials). The rationale for including all respondents in the modeling, even those who failed a quality assurance check, is detailed in Appendix 10 in Supplementary Materials.

**Table 2:** Logit and mixed logit models.

Variables	Logit model with interactions	Parameters	Mixed logit model 1: All normal	Parameters	Mixed logit model 2: Normal and log-normal
Alternative-specific constant (ref: Surgery A or B)					
Medication opt-out	0.638^∗∗∗^ (0.142)	Mean	−0.565^∗^ (0.277)	Mean	−0.688^∗^ (0.278)
		SD	4.016^∗∗∗^ (0.331)	SD	3.944^∗∗∗^ (0.320)
Improvement of Quality of life (ref: Mild)					
Moderate	0.430^∗∗∗^ (0.067)	Mean	0.548^∗∗∗^ (0.097)	Mean log-normal	−0.662^∗∗^ (0.226)
				Mean†	0.586^∗∗^
		SD	−0.276 (0.334)	SD log-normal	−0.506^∗^ (0.242)
				SD	−0.317^∗^
High	0.591^∗∗∗^ (0.079)	Mean	0.876^∗∗∗^ (0.120)	Mean log-normal	−0.236 (0.176)
				Mean	0.936
		SD	0.614^∗∗∗^ (0.180)	SD log-normal	0.582^∗∗∗^ (0.173)
				SD	0.594^∗∗∗^
Daily bowel frequency					
Linear bowel frequency	−0.181^∗∗∗^ (0.019)	Mean	−0.291^∗∗∗^ (0.030)	Mean log-normal	−1.381^∗∗∗^ (0.127)
				Mean	−0.307^∗∗∗^
		SD	0.202^∗∗∗^ (0.047)	SD log-normal	0.634^∗∗∗^ (0.085)
				SD	0.216^∗∗∗^
Bowel urgency (Ref: Need to hurry to the toilet)					
Can delay for up to 15 mins	0.948^∗∗∗^ (0.092)	Mean	1.358^∗∗∗^ (0.147)	Mean log-normal	0.290^∗∗^ (0.108)
				Mean	1.337^∗∗^
		SD	−0.022 (0.049)	SD log-normal	0.009 (0.032)
				SD	−0.012
Can delay as long as needed	1.314^∗∗∗^ (0.096)	Mean	1.887^∗∗∗^ (0.164)	Mean log-normal	0.523^∗∗∗^ (0.102)
				Mean	1.972^∗∗∗^
		SD	−0.934^∗∗∗^ (0.115)	SD log-normal	−0.559^∗∗∗^ (0.069)
				SD	−1.194^∗∗∗^
Having a stoma (Ref: No stoma)					
Temporary stoma	−0.217^∗^ (0.107)	Mean	−0.284^∗^ (0.134)	Mean	−0.315^∗^ (0.135)
		SD	0.311 (0.335)	SD	0.361 (0.272)
Interaction term: Temporary stoma # Male shift	−0.096 (0.175)	Interaction	−0.194 (0.194)	Interaction	−0.199 (0.197)
Reversible stoma	−0.361^∗∗∗^ (0.105)	Mean	−0.556^∗∗∗^ (0.134)	Mean	−0.553^∗∗∗^ (0.138)
		SD	0.467^∗^ (0.195)	SD	0.496^∗∗^ (0.188)
Interaction term: Reversible stoma # Male shift	−0.374^∗^ (0.169)	Interaction	−0.564^∗∗^ (0.193)	Interaction	−0.602^∗∗^ (0.199)
Permanent stoma	−1.197^∗∗∗^ (0.163)	Mean	−2.153^∗∗∗^ (0.292)	Mean	−2.154^∗∗∗^ (0.289)
		SD	−1.521^∗∗∗^ (0.253)	SD	−1.409^∗∗∗^ (0.259)
Interaction term: Permanent stoma # Male shift	−0.532^∗^ (0.239)	Interaction	−0.890^∗^ (0.359)	Interaction	−1.003^∗∗^ (0.362)
10-year risk of needing a permanent stoma					
Linear 10-year risk of stoma	−0.017^∗∗∗^ (0.004)	Mean	−0.038^∗∗∗^ (0.007)	Mean log-normal	−3.927^∗∗∗^ (0.352)
				Mean	−0.041^∗∗∗^
		SD	0.031^∗^ (0.015)	SD log-normal	−1.213^∗∗∗^ (0.219)
				SD	0.075^∗∗∗^
Risk of short-term complications					
Linear short-term complications	−0.014^∗∗∗^ (0.003)	Mean	−0.026^∗∗∗^ (0.005)	Mean log-normal	−4.641^∗∗∗^ (0.370)
				Mean	−0.039^∗∗∗^
		SD	−0.024^∗∗^ (0.009)	SD log-normal	1.670^∗∗∗^ (0.150)
				SD	0.152^∗∗∗^
Fertility (Ref: Women:19%; Men:0%)					
Women:25%; Men:0%	0.059 (0.105)	Mean	0.048 (0.146)	Mean	0.037 (0.147)
		SD	0.300 (0.241)	SD	−0.203 (0.476)
Interaction term: Women:25%; Men:0% # Plan to have children shift	−0.468^∗^ (0.203)	Interaction	−0.575^∗^ (0.251)	Interaction	−0.586^∗^ (0.259)
Women:40%; Men:0%	−0.029 (0.096)	Mean	0.010 (0.126)	Mean	−0.033 (0.130)
		SD	−0.024 (0.064)	SD	0.043 (0.143)
Interaction term: Women:40%; Men:0% # Plan to have children shift	−0.554^∗^ (0.219)	Interaction	−0.801^∗∗^ (0.261)	Interaction	−0.836^∗∗^ (0.276)
Women:43%; Men:15%	−0.107 (0.088)	Mean	−0.201 (0.128)	Mean	−0.214 (0.129)
		SD	−0.374 (0.299)	SD	−0.310 (0.350)
Interaction term: Women:43%; Men:15% # Plan to have children shift	−0.507^∗^ (0.202)	Interaction	−0.736^∗∗^ (0.270)	Interaction	−0.746^∗∗^ (0.277)
Observations	3150		3150		3150
Log-likelihood	−3019.16		−2253.28		−2236.9
BIC	6221.39		4780.43		4747.67
AIC	6078.31		4574.55		4541.79

*Note.* Standard errors, which are cluster-robust to account for multiple responses, are presented in parentheses.

AIC indicates Akaike information criterion; BIC, Bayesian information criterion.

∗*P* < .05, ∗∗ *P* < .01, ∗∗∗ *P* < .001 (2-sided cluster-robust *P* values).

† The mean and SD of the parameters were calculated using exp(m + sˆ2/2) and mean√(exp(sˆ2) − 1), respectively.^5^

### Conditional Distributions

**Figure 2:**
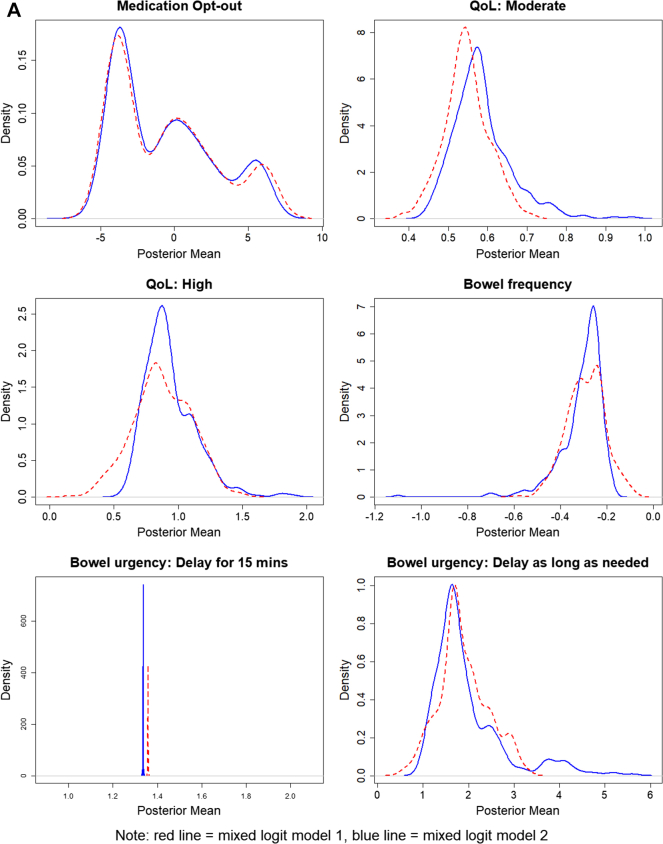

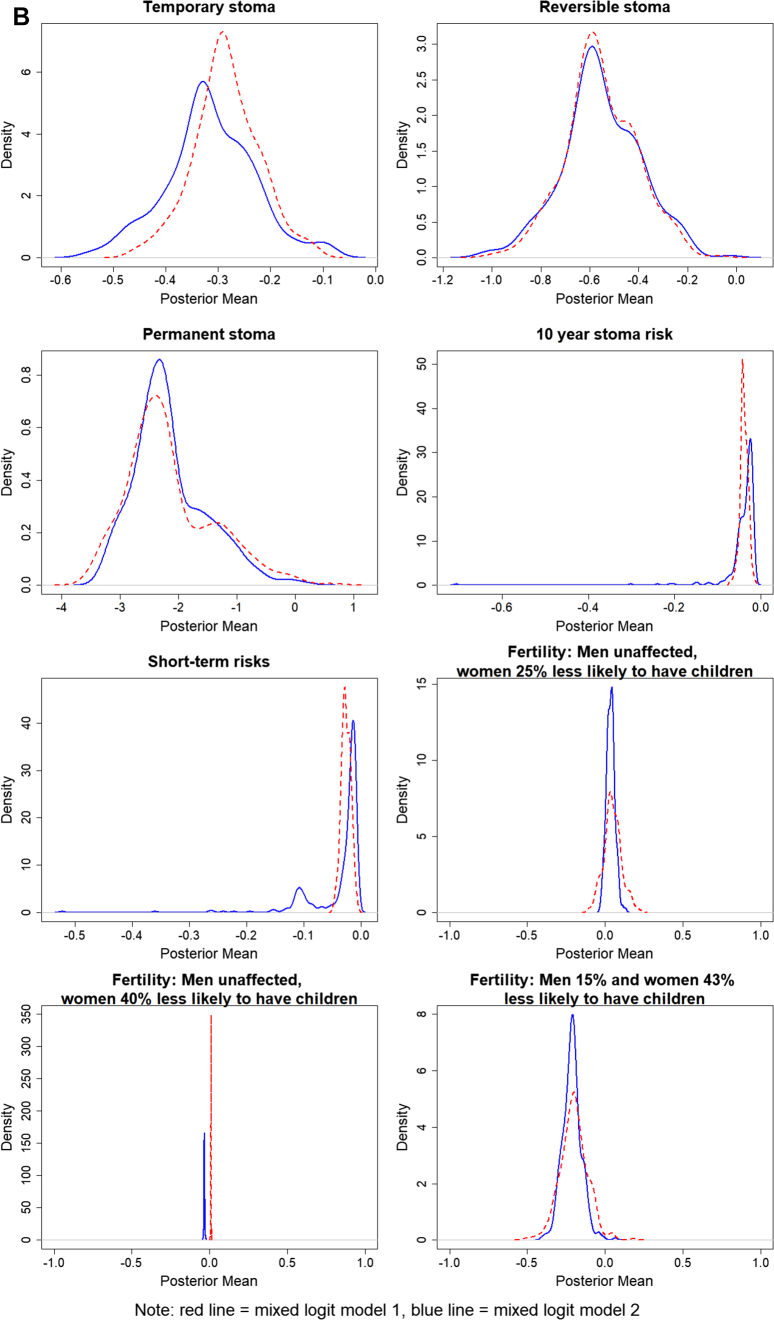
(A) Conditional distributions of the 2 mixed logit models. (B) Conditional distributions of the 2 mixed logit models.

Figure 2A and B depict the density plots of the conditional distributions that were estimated from the 2 mixed logit models in Table 2. Conditional distributions from mixed logit model 1, in which all the attributes are normally distributed, are shown with the dashed red line. The blue line shows the conditional distributions from the mixed logit model 2, in which the following attributes were log-normally distributed: quality of life, bowel frequency, bowel urgency, 10-year stoma risk, short-term risks, and the remaining distributions were specified to be normal. Overall, the conditional distributions are relatively similar between the 2 models, although there are some exceptions. First, almost all the attributes that were specified to be log-normal have a long tail. This is not surprising given that a long tail is a property of the log-normal distribution and has been previously reported in the DCE literature.^5^^,^^36^^,^^37^ Second, the conditional distributions for the 2 attributes—bowel frequency (delay 15 mins) and fertility (woman 40%, men 0%)—did not have any overlap between the 2 mixed logit models. However, these distributions had very little variance, and the difference between the means of the distributions is very small (∼0.02). Third, the medication opt-out shows a multimodal distribution with 3 noticeably different groups of people: those who prefer medication with positive coefficients, those who are indifferent centered around 0, and those who do not prefer medication with negative coefficients.

**Figure 3:**
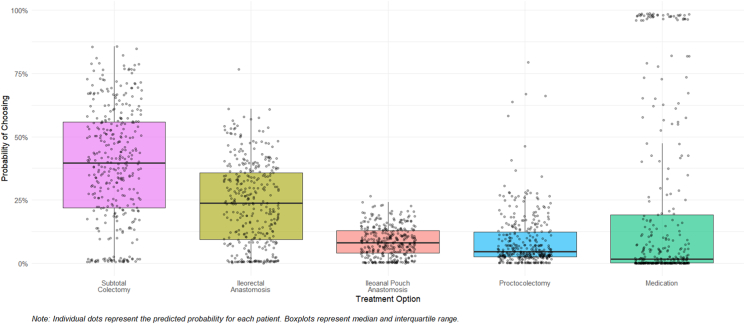
Heterogeneity in conditional choice probabilities.

Table 3 presents the conditional probability (individual-specific uptake rates) for different treatment scenarios for 3 randomly selected individuals from the sample, conditioned on the individuals’ observed choices. The scenarios were constructed with input from clinicians. These results show that making use of conditional densities can estimate choice probabilities that are more specific to individuals. For example, patient A overwhelmingly prefers medication (97.98% [96.44-99.10%]), whereas patient B is unlikely to choose medication (0.09% [0.03-0.16%]) and shows a preference for ileorectal anastomosis (52.64% [42.97-77.72%]). Patient C shows a preference for either subtotal colectomy (28.71% [14.84-55.46%]) or ileorectal anastomosis (28.32% [10.86-38.67%]), but the large, overlapping confidence intervals indicate considerable uncertainty in distinguishing between these options (see Appendix 11 in Supplementary Materials). Based on predicted probabilities across the population, subtotal colectomy was the preferred option, followed by ileorectal anastomosis (Fig. 3).

**Table 3:** Conditional probabilities for different treatment scenarios.

Treatment features	Subtotal colectomy	Proctocolectomy	Ileoanal pouch anastomosis	Ileorectal anastomosis	Medication
Improvement of quality of life	High	High	Moderate	Moderate	Low
Daily bowel frequency	4 times	4 times	5 times	3 times	7 times
Bowel urgency	Can delay as long as needed	Can delay as long as needed	Can delay for up to 15 mins	Can delay for up to 15 mins	Need to hurry
Having a stoma	Reversible stoma	Permanent stoma	Temporary stoma (no stoma after 12 weeks)	No stoma	No stoma
Risk of needing a permanent stoma at 10 years	5%	No risk	13%	21%	No risk
Risk of short-term complications	10%	10%	9%	10%	No risk
Fertility	Women: 19%; Men: 0%	Women: 40%; Men: 0%	Women: 43%; Men: 15%	Women: 25%; Men: 0%	No risk
Choice probability for a randomly selected person A [95% CI]∗	0.77% [0.32, 1.34]	0.14% [0.05, 0.29]	0.35% [0.15, 0.69]	0.76% [0.30, 1.45]	97.98% [96.44, 99.10]
Choice probability for a randomly selected person B [95% CI]	20.76% [8.95, 28.89]	12.07% [4.24, 20.94]	14.44% [6.17, 18.37]	52.64% [42.97, 77.72]	0.09% [0.03, 0.16]
Choice probability for a randomly selected person C [95% CI]	28.71% [14.84, 55.46]	7.04% [2.95, 13.62]	16.76% [8.41, 32.41]	28.32% [10.86, 38.67]	19.17% [12.24, 23.12]

∗ 95% CI were calculated using parametric bootstrapping. We generated 1000 draws from the coefficient distributions. For each draw, we generated individual-specific parameters, which we then used to calculate individual-specific probabilities.

## Discussion

This study assessed preference heterogeneity for surgical treatments among patients with UC. The results show that there is significant preference heterogeneity for most attributes included in the DCE, with the exception of the fertility attribute. Although the multinomial logit model found a positive preference for the opt-out, the mixed logit models revealed a significant negative preference with a multimodal conditional distribution. This multimodal distribution showed 3 distinct groups: those who strongly prefer medication, those who are indifferent, and those who actively do not prefer medication. Relying solely on the mean preference estimate risks masking these essential underlying preference clusters.

A typical approach in DCE literature for managing observed heterogeneity involves applying interaction terms based on sociodemographic characteristics. In our models, the interaction term “planning to have children” fully explained the preference heterogeneity in the fertility attribute. Patients planning to have children showed a stronger negative preference for surgical options associated with higher fertility risks. For patients not planning to have children, the main fertility risk attribute had a coefficient near zero, indicating that the attribute did not influence their choices. A key consideration during DCE development was whether to include the fertility attribute because it was irrelevant for a subset of the population. Alternatively, an adaptive design could have been used to dynamically exclude irrelevant attributes based on initial respondent input. However, the results show that for whom the fertility attribute was personally not relevant, the attribute had virtually no impact on their decision but was highly relevant to a subgroup of patients. Although this interaction term fully accounted for the heterogeneity in the fertility attribute, this is not always observed in our model.

More commonly, an interaction term only partially accounts for taste differences found in the data. For example, the significant gender interaction term showed that male patients held an even stronger negative preference for both permanent and reversible stomas than female patients. This finding suggests that, within our sample, men may place a higher priority on reconstructive surgeries that avoid a stoma. Although the interaction term accounted for some of the heterogeneity, using a mixed logit model was necessary to capture the remaining unobserved heterogeneity for this attribute. This combined approach provided a better fit to the data compared with a multinomial logit model with interactions alone.

Although the mixed logit models describe the population distribution, simply knowing that coefficients vary in a distribution is not its major advantage. Instead, one of the key benefits of mixed logit models is the post estimation analyses that can be done using this population distribution conditional on an individual’s specific response pattern. This article details how conditional mean estimates can be calculated and used to estimate individual-specific conditional probabilities. Our results demonstrated the utility of these conditional probabilities in generating highly specific predictive results. For example, person A had a 97.98% probability of choosing medication, whereas person B had only a 0.09% probability. This discriminatory power demonstrates the capacity of mixed logit models to move beyond average preferences and estimate individual-specific probabilities tailored to a person’s observed choices in the DCE. These individual-specific conditional probabilities could be used in preference-sensitive decisions, allowing patients and the healthcare team to make a shared decision aligned with the patient’s unique preferences.^38^ The next phase of this work will involve incorporating these individual-specific results directly into a patient decision aid.^39^ This tool integrates the DCE tasks to provide real-time outputs. Based on the choices a patient makes, the tool provides tailored results such as individual-specific conditional probabilities and relative importance scores. The intended purpose of such a tool is to facilitate shared decision making by empowering patients to clearly articulate their preferences with their healthcare professional to make an informed, values-based treatment decision.

According to the literature, the conditional parameter estimates from the mixed logit models enable the identification of outliers and the estimation of more accurate choice probabilities.^40^^,^^41^ Empirical studies in health have compared DCE probability predictions to real-world choices.^42^ These studies find that advanced models (such as heteroskedastic multinomial logit or mixed logit) that account for variation in decision making significantly improve predictive accuracy. For example, one study on influenza vaccination found that when models accounted for both scale and preference heterogeneity, real-world choices were correctly predicted by the model for 91% of the respondents.^43^ Another study on latent tuberculosis treatment found that using individual-specific coefficients, derived from a mixed logit model using hierarchical Bayesian estimation, correctly predicted actual treatment decisions for 83% of participants.^44^ Although mixed logit models are frequently estimated in the health literature,^3^ the estimation of these conditional parameters remains uncommon.

A critical issue when specifying mixed logit models is the choice of the mixing distribution used to estimate the random coefficients (normal, log-normal, etc). The majority of DCEs in health use the normal distribution to estimate these models,^45^ but this can be problematic for some attributes due to potential sign violations. Alternatively, the log-normal distribution is used because it ensures coefficients have the expected sign (negative for adverse outcomes like risk). In our study, we compared 2 mixed logit specifications: one assuming all attributes were normally distributed and another incorporating log-normal distributions for several key attributes based on theoretical considerations (bowel frequency, short- and long- term risks). The attributes specified as log-normal exhibited the expected long tail in their conditional distribution plots. This long tail has previously been criticized for potentially producing unrealistic marginal rates of substitution estimates.^37^ However, a visual comparison of the 2 specifications showed that the conditional coefficient distributions were broadly similar across both models, although there were noticeable differences, especially in the tails of the risk attributes. Further research is needed to explore the potential influence of the long tails on conditional probabilities.

### Limitations

This study has some limitations. Eligibility was based on a self-reported diagnosis of UC without clinical verification, which may contribute to potential sampling error. However, participants who registered for this national UC registry were not remunerated for taking part; therefore, it is unlikely they were attempting to misrepresent their condition. Since the sample was recruited from a national registry, it is broadly representative of the UK UC population^46^ in terms of gender and surgery rate. While young adults are underrepresented compared with national data, our sample includes representation across all age categories. Although this may limit generalizability to the youngest patients, our analyses found that the only demographics to significantly influence preferences were gender and family planning status. Additionally, a small proportion (8.3%) of the study cohort had already undergone surgery for UC. While this group differs from the surgery-naïve population, feedback from the pilot phase indicated that these participants could successfully interpret and complete the hypothetical tasks. To ensure decision-making consistency across the cohort, the survey instructed all participants to imagine a scenario (containing the symptom profile and decision context), regardless of their current surgical history.

Although the inclusion of 7 attributes is slightly above the average range of 4 to 6 used in the health DCE literature,^3^ the complexity of comparing multiple surgical options warranted the inclusion of a slightly higher number of attributes. The final set of attributes was selected through a robust, iterative process involving literature reviews and input from patients and healthcare professionals.

The method relied on to estimate conditional probabilities in this study was to use conditional mean coefficients directly in the logit formula. As detailed by Train, other approaches exist, such as incorporating the entire conditional density using simulation or accounting for the sampling variance of population parameters [12]. We used the parsimonious approach, which does not account for the distribution of coefficients around the conditional mean. Further research is needed to assess the impact of different estimation methods on these conditional probabilities. Additionally, reducing the DCE tasks from 12 to 9 could have increased the variance of the posterior distribution. Further research is needed to assess the optimal number of choice tasks required to improve prediction accuracy. It is important to clarify the nature of the individual-specific conditional probabilities discussed in this study. Although the mixed logit model provides these estimates for each person conditioned on their choices, these are still recovered from the overall population model and not from a model based on a single individual’s data. Furthermore, the external validity of the individual-specific conditional probabilities remains unexplored in this article. Although empirical studies in health have shown that accounting for heterogeneity can align DCE predictions with real-world choices,^42^ further research is needed to test the external validity of the conditional probabilities against real-world uptake.

## Conclusions

Our results demonstrate the value of using mixed logit models and their resultant conditional parameter estimates to analyze preferences for surgical treatments for patients with UC. Although multinomial logit models with interactions may suffice for estimating mean preferences, they fail to reveal the wide range of heterogeneity of preferences present in the data. The use of conditional probabilities enables the generation of choice probabilities that are tailored to individual respondents. These predictions can be used to inform shared decision making in preference-sensitive contexts, such as the choice of surgery for ulcerative colitis.

## Article and Author Information

**Authorship Confirmation:** All authors certify that they meet the ICMJE criteria for authorship.

**Funding/Support:** This work was funded by the NIHR Doctoral Fellowship (NIHR302489).

**Role of the Funder/Sponsor:** The funders played no role in the conduct of this study.

## Author Disclosures

Author disclosure forms can be accessed below in the Supplemental Material section. The views expressed herein are those of the authors and are not necessarily those of the funders. Dr Rowen is an editor for *Value in Health* and had no role in the peer-review process of this article.

## Supplemental Material

Supplemental Material

Author Disclosures
