# Supplementary material for: Beyond the Average: Modeling Individual-Specific Preferences for Ulcerative Colitis Surgery
Source: Value Health. 2026 Aug;29(8):1491–503. doi: 10.1016/j.jval.2026.02.010 (PMC13438707; doi:10.1016/j.jval.2026.02.010)
Supplement: Supplementary file 1 [file mmc2.pdf]

steven Brown

Discloser Identifier: 141362677
Disclosure Purpose: VIH-2025-1249 - Beyond the Average: Modelling Individual-Specific Preferences for Ulcerative Colitis Surgery.

Employment Information: Currently Employed

Summary of Interests

Company or Organization

Entity	Type		Relevant to this Disclosure
**sheffield Teaching hospitals**	Consultant	Current Employment	

Category: Consultant

Additional Questions

1. **Please select which of the following apply to each relationship or activity:**
   a. **Other Professional Activities - Consultant** sheffield Teaching hospitals

      Neither

2. **I confirm I have disclosed all direct support for the present manuscript (e.g. funding, provision of study materials, medical writing, article processing charges, etc.) There is no time limit for this item.**

   Yes

3. **Please indicate below whether in the past 36 months you have had any of the following interests that are topically related to the work reported in the manuscript.**
   a. **Employment (If you need to add an interest, please scroll to the top of the page, click "add interest" and select “Employment”)**

      No, I have no relevant interests of this type

   b. **Grants or contracts for research (If you need to add an interest, please scroll to the top of the page, click "add interest" and select “Grant/Contract”)**

      No, I have no relevant interests of this type

   c. **Payment for consulting (If you need to add an interest, please scroll to the top of the page, click "add interest" and select “Independent Contractor”)**

      No, I have no relevant interests of this type

   d. **Payments or honoraria for lectures, presentations, speakers bureaus, or educational events (If you need to add an interest, please scroll to the top of the page, click "add interest" and select "Independent Contractor" and include the correct information under "Consultant")**

      No, I have no relevant interests of this type

   e. **Payment for service on an advisory board (If you need to add an interest, please scroll to the top of the page, click "add interest" and select “Independent Contractor,” and choose “Other”)**

      No, I have no relevant interests of this type

   f. **Payment for participation Data and safety monitoring board (If you need to add an interest, please scroll to the top of the page, click "add interest" and select “Independent Contractor”)**

      No, I have no relevant interests of this type

   g. **Expert witness testimony (If you need to add an interest, please scroll to the top of the page, click "add interest" and select “Independent Contractor”)**

      No, I have no relevant interests of this type

h. **Royalties from Patents, Trademarks, Copyrights or other intellectual property (If you need to add an interest, please scroll to the top of the page, click "add interest" and select the appropriate interest type)**

No, I have no relevant interests of this type

i. **Patents planned, issued, or pending, whether or not you receive royalties (If you need to add an interest, please scroll to the top of the page, click "add interest" and select "Patents")**

No, I have no relevant interests of this type

j. **Fiduciary Officer or Other Board Membership (If you need to add an interest, please scroll to the top of the page, click "add interest" and select "Fiduciary Officer")**

No, I have no relevant interests of this type

k. **Stock or stock options (If you need to add an interest, please scroll to the top of the page, click "add interest" and select the appropriate interest type)**

No, I have no relevant interests of this type

l. **Support for attending meetings or other travel (If you need to add an interest, please scroll to the top of the page, click "add interest" and select "Travel")**

No, I have no relevant interests of this type

4. **Was any individual paid to provide professional writing assistance with this manuscript?**

No.

5. **Have you or your institution received equipment, materials, drugs, or services in direct support of the work in the manuscript (without time limit) not disclosed above?**

No.

6. **In the past 36 months, have you received equipment, materials, drugs, medical writing, gifts or other services from for-profit or not-for-profit third parties whose interests may be affected by the content of the manuscript not disclosed above?**

No.

7. **Are there other financial or non-financial interests that readers could perceive to have influenced, or that give the appearance of potentially influencing, what you wrote in the submitted work not disclosed above.**

No.

Certification

I certify that I have answered every question and the information provided in this disclosure is complete and accurate.

Convey Global Disclosure System | AAMC

Arne Hole

Dec 19, 2025 6:44 AM ET
ISPOR

Discloser Identifier: 55025261
Disclosure Purpose: VIH-2025-1249 - Beyond the Average: Modelling Individual-Specific Preferences for Ulcerative Colitis Surgery.

Employment Information: Currently Employed

Summary of Interests

Company or Organization

Entity	Type	Relevant to this Disclosure
**Universitat Jaume I**	Employment Current Employment	

Title: Professor of Economics

Additional Questions

1. **Please select which of the following apply to each relationship or activity:**
   a. **Employment** Universitat Jaume I

      The relationship is in direct support of the work reported in the manuscript anytime from when the work was conceived

2. **I confirm I have disclosed all direct support for the present manuscript (e.g. funding, provision of study materials, medical writing, article processing charges, etc.) There is no time limit for this item.**

   Yes

3. **Please indicate below whether in the past 36 months you have had any of the following interests that are topically related to the work reported in the manuscript.**
   a. **Employment (If you need to add an interest, please scroll to the top of the page, click "add interest" and select “Employment”)**

      Yes, as disclosed above

   b. **Grants or contracts for research (If you need to add an interest, please scroll to the top of the page, click "add interest" and select “Grant/Contract”)**

      No, I have no relevant interests of this type

   c. **Payment for consulting (If you need to add an interest, please scroll to the top of the page, click "add interest" and select “Independent Contractor”)**

      No, I have no relevant interests of this type

   d. **Payments or honoraria for lectures, presentations, speakers bureaus, or educational events (If you need to add an interest, please scroll to the top of the page, click "add interest" and select "Independent Contractor" and include the correct information under "Consultant")**

      No, I have no relevant interests of this type

   e. **Payment for service on an advisory board (If you need to add an interest, please scroll to the top of the page, click "add interest" and select “Independent Contractor,” and choose “Other”)**

      No, I have no relevant interests of this type

   f. **Payment for participation Data and safety monitoring board (If you need to add an interest, please scroll to the top of the page, click "add interest" and select “Independent Contractor”)**

      No, I have no relevant interests of this type

   g. **Expert witness testimony (If you need to add an interest, please scroll to the top of the page, click "add interest" and select “Independent Contractor”)**

      No, I have no relevant interests of this type

h. **Royalties from Patents, Trademarks, Copyrights or other intellectual property (If you need to add an interest, please scroll to the top of the page, click "add interest" and select the appropriate interest type)**

No, I have no relevant interests of this type

i. **Patents planned, issued, or pending, whether or not you receive royalties (If you need to add an interest, please scroll to the top of the page, click "add interest" and select "Patents")**

No, I have no relevant interests of this type

j. **Fiduciary Officer or Other Board Membership (If you need to add an interest, please scroll to the top of the page, click "add interest" and select "Fiduciary Officer")**

No, I have no relevant interests of this type

k. **Stock or stock options (If you need to add an interest, please scroll to the top of the page, click "add interest" and select the appropriate interest type)**

No, I have no relevant interests of this type

l. **Support for attending meetings or other travel (If you need to add an interest, please scroll to the top of the page, click "add interest" and select "Travel")**

No, I have no relevant interests of this type

4. **Was any individual paid to provide professional writing assistance with this manuscript?**

   No.

5. **Have you or your institution received equipment, materials, drugs, or services in direct support of the work in the manuscript (without time limit) not disclosed above?**

   No.

6. **In the past 36 months, have you received equipment, materials, drugs, medical writing, gifts or other services from for-profit or not-for-profit third parties whose interests may be affected by the content of the manuscript not disclosed above?**

   No.

7. **Are there other financial or non-financial interests that readers could perceive to have influenced, or that give the appearance of potentially influencing, what you wrote in the submitted work not disclosed above.**

   No.

Certification

I certify that I have answered every question and the information provided in this disclosure is complete and accurate.

Convey Global Disclosure System | AAMC

Donna Rowen

Discloser Identifier: 55032238
Disclosure Purpose: VIH-2025-1249 - Beyond the Average: Modelling Individual-Specific Preferences for Ulcerative Colitis Surgery.

Employment Information: Currently Employed

Summary of Interests

Company or Organization

Entity	Type	Relevant to this Disclosure
**EuroQol Research Foundation**	Other	Yes
**Category:** Other		
**EuroQol Research Foundation**	Travel	Yes
**Horizon 2020 Framework Programme**	Grant / Contract	
**Innovative Medicines Initiative 2 Joint Undertaking**	Grant / Contract	
**Innovative Medicines Initiative 2 Joint Undertaking**	Grant / Contract	
**University of Sheffield**	Employment Current Employment	Yes
**Title:** Professor of Helath Economics		

Intellectual Property

Type	Is Licensed	Relevant to this Disclosure
**Other Intellectual Property - Receive developer royalties for SF-6Dv2, via my e**	-	Yes

Description: Receive developer royalties for SF-6Dv2, via my employer University of Sheffield, who own copyright

Type: Royalties

Additional Questions

1. **Please select which of the following apply to each relationship or activity:**
    a. **Other Professional Activities - Other** EuroQol Research Foundation

       The relationship is outside the work reported in the manuscript but topically related and within the past 36 months

    b. **Employment** University of Sheffield

       The relationship is in direct support of the work reported in the manuscript anytime from when the work was conceived

    c. **Grant / Contract** Horizon 2020 Framework Programme

       Neither

    d. **Grant / Contract** Innovative Medicines Initiative 2 Joint Undertaking

       Neither

    e. **Grant / Contract** Innovative Medicines Initiative 2 Joint Undertaking

       Neither

    f. **Travel** EuroQol Research Foundation

       The relationship is outside the work reported in the manuscript but topically related and within the past 36 months

    g. **Other Intellectual Property** Receive developer royalties for SF-6Dv2, via my employer University of Sheffield, who own copyright

The relationship is outside the work reported in the manuscript but topically related and within the past 36 months

2. **I confirm I have disclosed all direct support for the present manuscript (e.g. funding, provision of study materials, medical writing, article processing charges, etc.) There is no time limit for this item.**

   Yes

3. **Please indicate below whether in the past 36 months you have had any of the following interests that are topically related to the work reported in the manuscript.**

   a. **Employment (If you need to add an interest, please scroll to the top of the page, click "add interest" and select “Employment”)**

      Yes, as disclosed above

   b. **Grants or contracts for research (If you need to add an interest, please scroll to the top of the page, click "add interest" and select “Grant/Contract”)**

      Yes, as disclosed above

   c. **Payment for consulting (If you need to add an interest, please scroll to the top of the page, click "add interest" and select “Independent Contractor”)**

      No, I have no relevant interests of this type

   d. **Payments or honoraria for lectures, presentations, speakers bureaus, or educational events (If you need to add an interest, please scroll to the top of the page, click "add interest" and select "Independent Contractor" and include the correct information under "Consultant")**

      No, I have no relevant interests of this type

   e. **Payment for service on an advisory board (If you need to add an interest, please scroll to the top of the page, click "add interest" and select “Independent Contractor,” and choose “Other”)**

      No, I have no relevant interests of this type

   f. **Payment for participation Data and safety monitoring board (If you need to add an interest, please scroll to the top of the page, click "add interest" and select “Independent Contractor”)**

      No, I have no relevant interests of this type

   g. **Expert witness testimony (If you need to add an interest, please scroll to the top of the page, click "add interest" and select “Independent Contractor”)**

      No, I have no relevant interests of this type

   h. **Royalties from Patents, Trademarks, Copyrights or other intellectual property (If you need to add an interest, please scroll to the top of the page, click "add interest" and select the appropriate interest type)**

      Yes, as disclosed above

   i. **Patents planned, issued, or pending, whether or not you receive royalties (If you need to add an interest, please scroll to the top of the page, click "add interest" and select “Patents”)**

      No, I have no relevant interests of this type

   j. **Fiduciary Officer or Other Board Membership (If you need to add an interest, please scroll to the top of the page, click "add interest" and select “Fiduciary Officer”)**

      No, I have no relevant interests of this type

   k. **Stock or stock options (If you need to add an interest, please scroll to the top of the page, click "add interest" and select the appropriate interest type)**

      No, I have no relevant interests of this type

   l. **Support for attending meetings or other travel (If you need to add an interest, please scroll to the top of the page, click "add interest" and select “Travel”)**

      Yes, as disclosed above

4. **Was any individual paid to provide professional writing assistance with this manuscript?**

   No.

5. **Have you or your institution received equipment, materials, drugs, or services in direct support of the work in the manuscript (without time limit) not disclosed above?**

   No.

6. **In the past 36 months, have you received equipment, materials, drugs, medical writing, gifts or other services from for-profit or not-for-profit third parties whose interests may be affected by the content of the manuscript not disclosed above?**

   No.

7. **Are there other financial or non-financial interests that readers could perceive to have influenced, or that give the appearance of potentially influencing, what you wrote in the submitted work not disclosed above.**

   No.

Certification

I certify that I have answered every question and the information provided in this disclosure is complete and accurate.

Convey Global Disclosure System | AAMC

Nyantara Wickramasekera

Discloser Identifier: 65499743
Disclosure Purpose: VIH-2025-1249 - Beyond the Average: Modelling Individual-Specific Preferences for Ulcerative Colitis Surgery.

Employment Information: Currently Employed

Summary of Interests

Company or Organization

Entity	Type	Relevant to this Disclosure
**National Institute for Health Research**	Grant / Contract	Yes
**University of Sheffield**	Employment Current Employment	
**Title:** Research Fellow		

Additional Questions

1. **Please select which of the following apply to each relationship or activity:**
   a. **Employment** University of Sheffield

      The relationship is in direct support of the work reported in the manuscript anytime from when the work was conceived

   b. **Grant / Contract** National Institute for Health Research

      The relationship is outside the work reported in the manuscript but topically related and within the past 36 months

2. **I confirm I have disclosed all direct support for the present manuscript (e.g. funding, provision of study materials, medical writing, article processing charges, etc.) There is no time limit for this item.**

   Yes

3. **Please indicate below whether in the past 36 months you have had any of the following interests that are topically related to the work reported in the manuscript.**
   a. **Employment (If you need to add an interest, please scroll to the top of the page, click "add interest" and select "Employment")**

      Yes, as disclosed above

   b. **Grants or contracts for research (If you need to add an interest, please scroll to the top of the page, click "add interest" and select "Grant/Contract")**

      Yes, as disclosed above

   c. **Payment for consulting (If you need to add an interest, please scroll to the top of the page, click "add interest" and select "Independent Contractor")**

      No, I have no relevant interests of this type

   d. **Payments or honoraria for lectures, presentations, speakers bureaus, or educational events (If you need to add an interest, please scroll to the top of the page, click "add interest" and select "Independent Contractor" and include the correct information under "Consultant")**

      No, I have no relevant interests of this type

   e. **Payment for service on an advisory board (If you need to add an interest, please scroll to the top of the page, click "add interest" and select "Independent Contractor," and choose "Other")**

      No, I have no relevant interests of this type

   f. **Payment for participation Data and safety monitoring board (If you need to add an interest, please scroll to the top of the page, click "add interest" and select "Independent Contractor")**

      No, I have no relevant interests of this type

g. **Expert witness testimony (If you need to add an interest, please scroll to the top of the page, click "add interest" and select "Independent Contractor")**

No, I have no relevant interests of this type

h. **Royalties from Patents, Trademarks, Copyrights or other intellectual property (If you need to add an interest, please scroll to the top of the page, click "add interest" and select the appropriate interest type)**

No, I have no relevant interests of this type

i. **Patents planned, issued, or pending, whether or not you receive royalties (If you need to add an interest, please scroll to the top of the page, click "add interest" and select "Patents")**

No, I have no relevant interests of this type

j. **Fiduciary Officer or Other Board Membership (If you need to add an interest, please scroll to the top of the page, click "add interest" and select "Fiduciary Officer")**

No, I have no relevant interests of this type

k. **Stock or stock options (If you need to add an interest, please scroll to the top of the page, click "add interest" and select the appropriate interest type)**

No, I have no relevant interests of this type

l. **Support for attending meetings or other travel (If you need to add an interest, please scroll to the top of the page, click "add interest" and select "Travel")**

No, I have no relevant interests of this type

4. **Was any individual paid to provide professional writing assistance with this manuscript?**

   No.

5. **Have you or your institution received equipment, materials, drugs, or services in direct support of the work in the manuscript (without time limit) not disclosed above?**

   No.

6. **In the past 36 months, have you received equipment, materials, drugs, medical writing, gifts or other services from for-profit or not-for-profit third parties whose interests may be affected by the content of the manuscript not disclosed above?**

   No.

7. **Are there other financial or non-financial interests that readers could perceive to have influenced, or that give the appearance of potentially influencing, what you wrote in the submitted work not disclosed above.**

   No.

Certification

I certify that I have answered every question and the information provided in this disclosure is complete and accurate.
