# Supplementary material for: Beyond the Average: Modeling Individual-Specific Preferences for Ulcerative Colitis Surgery
Source: Value Health. 2026 Aug;29(8):1491–503. doi: 10.1016/j.jval.2026.02.010 (PMC13438707; doi:10.1016/j.jval.2026.02.010)
Supplement: Supplementary file 2 [file mmc1.docx]

## Appendix Table 1: DIRECT Checklist

| **Item** | **Description of Reporting Requirement** | **Page** |
| --- | --- | --- |
| 1 | Describe the real-world context and decision-maker that the hypothetical choice context seeks to replicate or inform. | P1 |
| 2 | Provide a rationale for using a DCE to answer the research question. | P2 |
| 3 | Describe how attributes and levels were derived (e.g., literature review, interviews, focus groups, expert input). | P2 |
| 4 | Provide the final list of attributes and levels. | Appendix 3 |
| 5 | Report the number of alternatives per choice set and whether they were labeled or unlabeled. | P2 |
| 6 | Describe response options (e.g., forced choice, opt-out, status quo). | P2-3 |
| 7 | Describe the type of experimental design (e.g., orthogonal, D-efficient, Bayesian efficient, partial profile). | P2 |
| 8 | Describe which effects are identified in the design (e.g., main effects, higher order interactions, functional form). | P2 |
| 9 | Describe the number of choice sets, blocks, and choice sets per block. | P2 and Appendix 4 |
| 10 | Indicate how the experimental design was obtained (software, catalogue, other). | P2 and Appendix 4 |
| 11 | Provide a sample choice set and the instructions and background information given to respondents (e.g., as an appendix). | P3 and Appendix 5 |
| 12 | Report any randomization (e.g., choice set order, attribute order, alternative order, framing effects). | Appendix 4 |
| 13 | Describe what was checked in piloting (e.g., understanding, respondent burden, timing, wording). | P2 |
| 14 | Report whether pilot information was used to update the experimental design (e.g., priors, functional form) or survey design. | Appendix 4 |
| 15 | Report respondent inclusion/exclusion criteria. | P2 |
| 16 | Describe how data were collected (e.g., mail, personal interview, web survey). | P2-4 |
| 17 | Report the response rate or cooperation rate, if possible. | P4 |
| 18 | Report the final sample size and how the sample size was determined. | P4 |
| 19 | Describe respondent characteristics and representativeness of the target population, if known. | P4, P11 |
| 20 | Indicate coding of data (e.g., effects, dummy, continuous) including definitions. | P4 |
| 21 | Report whether any respondents were removed and why (e.g., suspected fraudulent responses, rationality tests). | P5 |
| 22 | Provide the rationale for model choice (e.g., conditional logit, mixed logit, latent class) and assumptions (e.g., error variance). | P4 -5 |
| 23 | Report model specification. | P5-7 |
| 24 | Report model performance and goodness of fit (if comparing models). | P7 |
| 25 | Describe methods used for analysis of results (e.g., marginal rate of substitution, attribute importance, welfare gain). | P4, |
| 26 | Report measures of precision for outputs of interest (e.g., confidence intervals) and how these were derived. | P4, P10 |

## Appendix Table 2: Attribute selection process

|  | **Surgery attribute list identified from the literature** | **References** | **Changes made after iterative rounds of discussions with the clinical team (n=4; 2 medical and 2 surgical)** | **Change made after patient interviews (n=10)** |
| --- | --- | --- | --- | --- |
| 1 | Stoma | (1-6) | √ | √ |
| 2 | Quality of life/pain/sleep/mood/usual activities | (1-6) | √ | √ |
| 3 | Functional outcomes/disease activity/ | (1-6) | √ | √ |
| 4 | Urgency | (3, 4) | √ | √ |
| 5 | Increased risk of colorectal cancer | (3, 5, 7, 8) | √ | X Consistently ranked as the least important attribute because of negligible average risk differences between surgeries and was deemed suitable for removal. Providing a personalised cancer risk, a function of residual colorectum and mucosal inflammation duration, rendered its inclusion operationally infeasible. |
| 6 | Impact on fertility/ fertility reduction | (1, 3, 9) | √ Merged as they are related | √ |
| 7 | Sexual function | (4, 9) |  |  |
| 8 | Long Term Treatment failure | (7, 9) | √ These complications were too numerous to include as separate attributes, clinicians recommended to group these to short term and long term: acute short term severe complications (Clavien-Dindo III-IV) that require further surgical intervention | √ |
| 9 | Bowel obstruction/illeus | (10) |  |  |
| 10 | Anastomotic leak | (10) |  |  |
| 11 | Pouchitis | (10) |  |  |
| 12 | Faecal incontinence | (9) |  |  |
| 13 | Parastomal hernia | (10) |  |  |
| 14 | Increased risk of serious infection | (1, 3, 5, 7) |  |  |
| 15 | Dietary Impact | (9) | x | X Recognised as important, but ultimately not selected for including in the DCE |
| 16 | Practicalities of caring of a stoma | (9, 10) | √ | X Patients reported that the existing stoma information was adequate. |
| 17 | Ongoing medication and surveillance needed after surgery | (9) | √ | X Recognised as important, but ultimately not selected for including in the DCE |
| 18 | Number of operations needed | (1, 4, 9, 10) | √ | X Important but highly correlated with other attributes, so have highlighted this in the stoma attribute where applicable. |
| 19 | Laparoscopic vs open | (1) | X Too individual context specific to include in DCE. | x |
| 20 | Time spent in hospital after surgery/ time off work / recovery time | (9) | √ | √ Included in the surgery label description but not as a separate attribute because all surgeries roughly had the same recovery period. |
| Note: √ = attribute retained, X = attribute excluded | | | | |

## Appendix Table 3: Attributes included in the DCE

| **Attributes** | **Levels** |
| --- | --- |
| Daily bowel frequency (B) | 2 times  3 times  4 times  5 times  6 times  7 times (ref) |
| Bowel urgency (I) | Can delay as long as needed  Can delay for up to 15 mins  Need to hurry to the toilet (ref) |
| Improvement of quality of life (QoL) (Q) | Mild (some pain and fatigue, sleep and usual activities disrupted) (ref)  Moderate (a little pain and fatigue, sleep well, can do most usual activities)  High (no pain and fatigue, sleep well, can do all usual activities) |
| Having a stoma (S) | Permanent stoma  Reversible stoma  Temporary stoma (no stoma after 12 weeks)  No stoma (ref) |
| Risk of needing a permanent stoma at 10 years (L) | No risk (ref)  5 out of 100 people (5%)  10 out of 100 people (10%)  20 out of 100 people (20%) |
| Risk of short-term complications (R) | 5 out of 100 people (5%) (ref)  10 out of 100 people (10%)  20 out of 100 people (20%)  30 out of 100 people (30%) |
| Fertility (compared to general population) (F) | Women are 43% less likely, men 15% less likely to have children.  Women are 40% less likely to have children, men's fertility is unaffected.  Women are 25% less likely to have children, men's fertility is unaffected.  Women are 19% less likely to have children, men's fertility is unaffected (ref) |

## Appendix 4: Ngene Design

A D-efficient fractional factorial design using directional priors was generated using the Ngene software. The design was optimised for a main-effects multinomial logit model. A fractional factorial design was chosen to avoid the unmanageable number of attribute combinations generated in a full factorial design. The Modified Federov algorithm was used to allow flexibility in relaxing attribute level balance (0.1 imbalance) and to impose constraints on the design. Two constraints were applied to ensure logical alternative generation: 1) co-occurrence of high quality of life and a need to hurry to the toilet was prohibited, and 2) the risk of needing a permanent stoma at 10 years was set to zero for alternatives with a permanent stoma. The final design, comprising 36 tasks divided into four blocks, was selected based on the lowest D-error score. Participants were randomly assigned to complete one block of nine tasks, with the display order of the choice sets randomised to mitigate potential ordering effects. We chose not to update directional priors following the pilot phase to maintain design robustness. Updating designs based on priors from the pilot carries a risk of design deficiency if the pilot priors are misspecified for the broader population (14).

## Appendix 5: Full survey

The DCE survey was hosted online using the Qualtrics platform. The survey consisted of five parts: sociodemographic data collection, an introduction defining attributes of the DCE task, nine DCE tasks, ulcerative colitis treatment history, and survey feedback (see fill survey text below). The introductory section provided detailed explanations of each attribute and included practice DCE tasks, with complexity increasing incrementally by the addition of one or two attributes per task. In this section, a scenario was constructed to frame the experiment. This description was important so that all patients could answer the task while considering the same context in which the DCE tasks were placed. The treatment history section incorporated the IBD-Control-8 disease activity questionnaire, a validated instrument widely used in the UK (13). To ensure data quality, several internal validity checks were incorporated, with feedback provided where appropriate. First, a risk comprehension test was included to test participants’ grasp of probabilities. This task contained two surgical profiles where one was unambiguously superior, with surgical complication risks illustrated using both icon arrays and percentages. Second, a “check your understanding” quiz question was used to verify respondents' comprehension of the attribute details provided on the previous screen. Third, Qualtrics fraud detection was activated to detect bots and duplicate entries.

**Online survey about patients’ preferences for surgical and medical treatments of ulcerative colitis**

This survey is open to people with a diagnosis of ulcerative colitis who are 16 and older and living in the UK. The online survey is expected to take around 15 minutes of your time. We will ask you questions about yourself including about your treatments for ulcerative colitis. Then we will also show you a series of different treatments and ask you to tell us which treatment you think is better. By taking part you will be providing valuable information which will be used to develop a patient decision tool to support shared decision making between people with ulcerative colitis and their doctor. This research is conducted for the fulfilment of a doctoral degree funded by the National Institute for Health Research.

**Will my taking part in this project be kept confidential?**

Yes. All the information that we collect about you will be kept strictly confidential and accessible only to members of the research team. You will not be identified in any reports or publications. The University of Sheffield will act as the Data Controller for this study. This means that the University is responsible for looking after your information and using it properly. At the end of the study, if you agree to us sharing the information you provide with other researchers, your anonymous data may be archived in a data repository for the benefit of future research.

**Do I have to take part? What are the possible disadvantages and risks of taking part?** There is no obligation to take part in the study. If you do take part, you are free to stop and exit the survey by closing the browser at any time. While we do not expect the survey questions to cause distress, if you feel that these questions may trigger upsetting thoughts or feelings, please do not proceed with this survey. We will be unable to withdraw your data after fully or partially completing the survey. This is because we do not collect any of your personal identifying information so we will not be able to identify and remove your data.

**What is the legal basis for processing my personal data?** The legal basis we are applying in order to process your personal data is that ‘processing is necessary for the performance of a task carried out in the public interest’ (Article 6(1)(e)). Further information can be found in the University’s Privacy Notice https://www.sheffield.ac.uk/govern/data-protection/privacy/general.  As we will be collecting some data that is defined in the legislation as more sensitive (information such as your age, gender, health and so on), we also need to let you know that we are applying the following condition in law: that the use of your data is ‘necessary for scientific or historical research purposes’ and Article 9(2)(j) processing is necessary for archiving purposes in the public interest, scientific or historical research purposes or statistical purposes.

**Who has ethically reviewed the project?** This project has been ethically approved via the University of Sheffield’s Ethics Review Procedure, as administered by the Sheffield Centre for Health and Related Research (SCHARR).

**What if something goes wrong and I wish to complain about the research?** If you are dissatisfied with any aspect of the research and wish to make a complaint, please contact Professor Donna Rowen at d.rowen@sheffield.ac.uk, in the first instance. If you feel your complaint has not been handled in a satisfactory way, you can contact the Dean of the Sheffield Centre for Health and Related Research (SCHARR) Dr Louise Preston at l.r.preston@sheffield.ac.uk. If the complaint relates to how your personal data has been handled, you can contact the University of Sheffield Data Protection Officer, Luke Thompson at dataprotection@sheffield.ac.uk.

Before starting this survey, please confirm the following:

- I have read and understood the information provided above
- I understand and agree that the University of Sheffield and other authorised researchers may use my anonymous survey responses in publications, reports, web pages, and other research outputs
- I give permission for the University of Sheffield to deposit my anonymous survey responses in a suitable online research archive so that it can be used in future research and learning. I understand it will not be possible to identify me from this information

End of Block: PIS

Start of Block: About you

**This survey consists of five parts:**

Part 1: About You

Part 2: Introduction to review the key terms used in this survey

Part 3: Preferences for surgical or medical treatment

Part 4: Your ulcerative colitis treatment history

Part 5: Survey feedback

What is your gender?

- Male
- Female
- Non-binary
- Prefer not to say

How old are you?

- 16-24 years old
- 25-34 years old
- 35-44 years old
- 45-54 years old
- 55-64 years old
- 65+ years old
- Prefer not to say

Which of the following best describes your main activity? Choose one, which is most applicable to you.

- In employment or self-employment
- Retired
- Looking after the home and family
- Taking care of a family member with chronic illness or disability
- Student
- Job-seeking (unemployed and looking for employment)
- Long-term sick or disabled
- Prefer not to say

What ethnic group best describes you?

- White
- Black / African / Caribbean / Black British
- Asian/Asian British
- Mixed / Multiple ethnic groups
- Prefer not to say

What is the highest level of education you have completed?

- Primary
- Secondary (GCSE/ O-Level)
- Further education (A-Level, BTEC)
- Bachelor's, Master's, Doctoral degree
- Prefer not to say

What is your marital status?

- Married/Partner
- Widowed
- Divorced/Separated
- Single
- Prefer not to say

Are you planning to have children?

- Yes
- No
- Prefer not to say

End of Block: About you

Start of Block: Attribute levels

We would like to understand what your priorities are when choosing between surgical and medical treatments for ulcerative colitis. We will give you information about the benefits and risks of different treatments. After carefully considering this information, you will be asked to select the treatment that you think would be best for you.

| Page Break |  |
| --- | --- |

In the question below you will be asked to choose between three treatment options: Surgery A, Surgery B and Medication. After either surgery, you would typically **stay in the hospital for about a week**, and you would need to **rest and recover at home for about 6 weeks** before returning to your usual activities. If you prefer not to have either Surgery A or Surgery B, you can choose to continue with medication.

Each treatment is described based on **seven** key features. Let's begin by reviewing the **first two** features:

1. **Daily bowel frequency**: The average number of times you are likely to use the toilet varies depending on the type of treatment you have. This can range from 2 to 7 times per day.
2. **Bowel urgency:** This describes how urgently you may need to use the toilet. After treatment, you may be able to delay as long as needed, delay for up to 15 minutes, or you may need to hurry to the toilet to avoid an accident.

Please select the treatment you would choose if these were the only choices available. Please make your selection based only on the information presented here.

Which treatment would you choose?

- Surgery A
- Surgery B
- Medication

The third feature is improvement of quality of life: following treatment, you may see improvements to your current symptoms that we asked you to imagine. You may experience less stomach pain and fatigue, sleep through the night, and find it easier to engage in your usual activities (work, school, sports, hobbies, and going out). The level of improvement to your quality of life can vary from:

- High (no pain and fatigue, sleep well, can do all usual activities)
- Moderate (a little pain and fatigue, sleep well, can do most usual activities)
- Mild (some pain and fatigue, sleep and usual activities disrupted)

Which treatment would you choose?

- Surgery A
- Surgery B
- Medication

The fourth feature is the possibility of **having a stoma**: some surgeries require the creation of a stoma. This involves bringing the end of the small bowel to the skin surface near the abdomen, where a bag is attached (also known as an ileostomy). The stoma bag collects stool and has an opening which helps you to empty the stool down the toilet, whilst you are sitting on the toilet. You would typically need to change the stoma bag every day. A stoma is not visible under clothing and does not stop you from doing your usual daily activities. For example, you can continue working, exercising, swimming, and stoma guards can be used during contact sports.

Depending on the surgical procedure, you may have one of the following:

**Permanent stoma**

This surgery will remove the colon and rectum. You will have a stoma that is irreversible, and the stoma will remain in place for the rest of your life. May require one or more surgeries.

**Reversible stoma**

During this single surgery, the colon is removed, and the rectum remains. A stoma will be created. You can undergo further surgery to remove the stoma if you decide that you do not want a stoma at a later stage.

**Temporary stoma (no stoma after 12 weeks)**

This surgery will remove the colon and rectum and reconstruct a new ‘rectum’ using the small bowel. When your bowel is fully healed, you will need another surgery to remove the temporary stoma. Once the stoma is removed, you will be able to go to the toilet as you did before.

**No stoma**

The surgery does not require the creation of a stoma. This surgery will remove the colon, and then connect the small bowel to the rectum. This may require one or more surgeries.

Check Your Understanding

Imagine that you had surgery needing a temporary stoma. Approximately how long will you likely have the stoma in place?

- Indefinitely
- about 12 weeks
- about 36 weeks

To go back and review the stoma information, click the back button below.

| Page Break |  |
| --- | --- |

Display This Question:

If Check Your Understanding Imagine that you had surgery needing a temporary stoma. Approximately ho... = about 12 weeks

Correct. Once the stoma is removed after about 12 weeks, you will be able to go to the toilet just as you did before.

Display This Question:

If Check Your Understanding Imagine that you had surgery needing a temporary stoma. Approximately ho... = Indefinitely

That is incorrect. Temporary stomas are used while the bowel heals, which takes about 12 weeks. Once the stoma is removed, you will be able to go to the toilet just as you did before.

Display This Question:

If Check Your Understanding Imagine that you had surgery needing a temporary stoma. Approximately ho... = about 36 weeks

That is incorrect. Temporary stomas are used while the bowel heals, which takes about 12 weeks. Once the stoma is removed, you will be able to go to the toilet just as you did before.

5. **Risk of needing a permanent stoma** at 10 years: there is a 5-20% chance you may need further surgery in the long term because of persistent symptoms (like diarrhoea and urgency) or because of early signs of bowel cancer.

Please select the option you would choose if these were the only choices available. Please make your selection based only on the information presented here.

Which treatment would you choose?

- Surgery A
- Surgery B
- Medication

Display This Question:

If 5. Risk of needing a permanent stoma at 10 years: there is a 5-20% chance you may need further su... = Surgery B

You have selected Surgery B: This means the surgery will not involve a stoma, but 10 years later there is a 20% chance of needing a permanent stoma.

Display This Question:

If 5. Risk of needing a permanent stoma at 10 years: there is a 5-20% chance you may need further su... = Surgery A

You have selected Surgery A: This means you will not have a stoma after your second operation, but 10 years later there is a 10% chance of needing a permanent stoma.

6. **Risk of short-term complications**: 1-2 weeks after either surgery there is a chance of experiencing serious side effects. These include leaking of stool due to imperfect healing, bleeding, injury to the bowel, a serious infection in the wound or chest, and clots in the lungs. Depending on the surgery you have, the risk of short-term complications varies from 5% to 30%. Let's review this information in a more visual way:

Each box shows a group of 100 people. The blue people in each box represent those who experienced serious side effects (such as leaking of stool due to imperfect healing, bleeding, or injury to the bowel, a serious infection in the wound or chest, and clots in the lungs) 1-2 weeks after the surgery, requiring further operations. The grey people represent those who did not experience any short-term complications.

| Page Break |  |
| --- | --- |

If these are the only two features that make the surgeries different, which surgery would you choose?

- Surgery A
- Surgery B

Display This Question:

If If these are the only two features that make the surgeries different, which surgery would you cho... = Surgery B

Correct. Surgery B presents a lower chance of complications immediately following the procedure and reduces the likelihood of needing a permanent stoma within 10 years.

Display This Question:

If If these are the only two features that make the surgeries different, which surgery would you cho... = Surgery A

That is incorrect. Surgery B has a lower risk of both short-term complications and the need for a permanent stoma within 10 years compared to Surgery A.

7. **Fertility:** Surgery may impact your fertility because the operation is close to the reproductive organs (also called the pelvis). This proximity means the surgery could reduce your chances of conceiving or fathering a child compared to the general population without ulcerative colitis.

Depending on the type of surgery:

- Women are 19% to 43% less likely to have children, compared to people in the general population without ulcerative colitis.
- Men may have little to no change (15% less likely) in their chances of having children, compared to people in the general population without ulcerative colitis.

Thank you for completing the introduction section.

The next section contains 9 treatment comparison tasks.

While all the tasks look similar, the information presented in the table is different, so please take the time to read all the information before making your choice.

The accuracy of our results depends on you carefully weighing the benefits and risks of each treatment.

End of Block: Attribute levels

Please select the option you would choose if these were the only choices available. Please make your selection based only on the information presented here.

Which treatment would you choose?

- Surgery A
- Surgery B
- Medication

Start of Block: treatment history

How long have you been diagnosed with Ulcerative Colitis?

- Less than 1 year
- 1 to 2 years
- 3 to 5 years
- 6 to 10 years
- More than 10 years

Have you had any of these medications in the **past**? (please choose all that applies)

- Steroids
- 5-Aminosalicylates/5-ASAs/Mesalamine/Mesalazine/Sulfasalazine
- Azathioprine/Mercaptopurine/Methotrexate/Tacrolimus
- Infliximab (Remicade, Flixabi, Inflectra, Remsima, and Zessly)
- Adalimumab (Humira, Idacio, Kromeya, Halimatoz, Hefiya, Amgevita, Hulio, Hyrimoz, and Imraldi)
- Vedolizumab (Entyvio)
- Ustekinumab (Stelara)
- Golimumab (Simponi)
- Certolizumab (Cimzia)
- Tofacitinib (Xeljanz)
- Upadacitinib (Rinvoq)
- Filgotinib (Jyseleca)
- Other, please specify: __________________________________________________
- None of the above

Have you had surgery to treat your ulcerative colitis?

- Yes
- No

Display This Question:

If Have you had surgery to treat your ulcerative colitis? = Yes

What kind of surgery did you have (please choose all that applies)

- Subtotal Colectomy (stoma)
- Panproctocolectomy/Proctocolectomy with ileostomy (permanent stoma)
- Ileoanal Pouch Anastomosis
- Ileorectal Anastomosis
- Other, please specify: __________________________________________________

Display This Question:

If Have you had surgery to treat your ulcerative colitis? = No

What is your **current** treatment for ulcerative colitis?

- Steroids
- 5-Aminosalicylates/5-ASAs/Mesalamine/Mesalazine/Sulfasalazine
- Azathioprine/Mercaptopurine/Methotrexate/Tacrolimus
- Infliximab (Remicade, Flixabi, Inflectra, Remsima, and Zessly)
- Adalimumab (Humira, Idacio, Kromeya, Halimatoz, Hefiya, Amgevita, Hulio, Hyrimoz, and Imraldi)
- Vedolizumab (Entyvio)
- Ustekinumab (Stelara)
- Golimumab (Simponi)
- Certolizumab (Cimzia)
- Tofacitinib (Xeljanz)
- Filgotinib (Jyseleca)
- Upadacitinib (Rinvoq)
- Other, please specify: __________________________________________________
- None of the above

Do you believe that:

|  | Yes | No | Not Sure |
| --- | --- | --- | --- |
| Your IBD has been well controlled in the past two weeks? |  |  |  |

Do you believe that:

|  | Yes | No | Not Sure | Not taking any treatment |
| --- | --- | --- | --- | --- |
| Your current treatment is useful in controlling your IBD |  |  |  |  |

In the past 2 weeks, did you:

|  | Yes | No | Not sure |
| --- | --- | --- | --- |
| Miss any planned activities because of IBD? (e.g. attending school/college, going to work or a social event) |  |  |  |
| Wake up at night because of symptoms of IBD? |  |  |  |
| Suffer from significant pain or discomfort? |  |  |  |
| Often feel lacking in energy (fatigued)? (by 'often' we mean more than half of the time) |  |  |  |
| Feel anxious or depressed because of your IBD? |  |  |  |
| Think you needed a change to your treatment? |  |  |  |

(IBD-Control Questionnaire © Keith Bodger, 2014)

Thinking about the first part of the survey where you were asked to choose between different treatments, please tell us how strongly you agree or disagree with each of the following statements.

|  | strongly disagree | disagree | uncertain | agree | strongly agree |
| --- | --- | --- | --- | --- | --- |
| I understood the questions about making choices between different treatment options |  |  |  |  |  |
| When choosing between different treatment options I needed more information than was provided |  |  |  |  |  |
| I found making a choice between different treatment options difficult |  |  |  |  |  |

Do you have any comments you would like to make in connection with this questionnaire?

________________________________________________________________

________________________________________________________________

## Appendix 6: Pilot version of the DCE task

## Appendix 7: Latent class model

The latent class model identified three distinct classes, with class 1 showing no significant preference for either medication or surgery, class 2 having a strong preference for medication and class 3 showing a strong preference for surgery. Notably, the sociodemographic characteristics included in the class membership function -gender and family planning -were not significant predictors of class assignment. While the latent class model offers a broad overview of preference heterogeneity, mixed logit models demonstrated better model fit (according to AIC and BIC scores) and provide a full distribution of tastes across the population.

|  | **Class 1 (51%)** | **Class 2 (20.5%)** | **Class 3 (28.5%)** |
| --- | --- | --- | --- |
| Medication opt-out | 0.185 (0.258) | 2.324^***^ (0.625) | -1.840^***^ (0.314) |
| **Improvement of Quality of life (ref: Mild)** | | | |
| Qol: Moderate | 0.325^*^ (0.142) | -0.315 (0.401) | 0.510^***^ (0.092) |
| Qol: High | 0.574^***^ (0.166) | -0.132 (0.430) | 0.823^***^ (0.120) |
| Linear bowel frequency | -0.281^***^ (0.042) | -0.175 (0.099) | -0.213^***^ (0.030) |
| **Bowel urgency (Ref: Need to hurry to the toilet)** | | | |
| Can delay for up to 15 mins | 0.985^***^ (0.200) | 0.985 (0.532) | 1.088^***^ (0.133) |
| Can delay as long as needed | 1.132^***^ (0.189) | 1.152^*^ (0.468) | 1.697^***^ (0.130) |
| **Having a stoma (Ref: No stoma)** | | | |
| Temporary stoma | -0.451^**^ (0.160) | -1.252^**^ (0.404) | -0.062 (0.119) |
| Reversible stoma | -0.950^***^ (0.160) | -1.742^***^ (0.454) | -0.406^***^ (0.109) |
| Permanent stoma | -1.973^***^ (0.313) | -2.569^**^ (0.816) | -1.630^***^ (0.201) |
| Linear 10-year risk of stoma | -0.039^***^ (0.009) | -0.038 (0.028) | -0.022^**^ (0.007) |
| Linear short-term complications | -0.036^***^ (0.007) | -0.027 (0.019) | -0.010^*^ (0.004) |
| **Fertility (Ref: Women:19%; Men:0%)** | | | |
| Women:25%; Men:0% | -0.006 (0.181) | -0.063 (0.389) | -0.160 (0.139) |
| Women:40%; Men:0% | -0.251 (0.173) | -0.874 (0.447) | -0.049 (0.127) |
| Women:43%; Men:15% | -0.430^*^ (0.186) | -1.011 (0.525) | -0.215 (0.129) |
| **Class membership** | | | |
| Male |  | 0.578 (0.306) | 0.099 (0.296) |
| Plan to have children |  | 0.569 (0.337) | 0.519 (0.326) |
| Constant |  | -1.028^***^ (0.279) | -1.101^***^ (0.230) |
| **Model fit** | | | |
| LL | -2268.91 | | |
| BIC | 4977.20 | | |
| AIC | 4633.82 | | |
| CAIC | 4867.00 | | |
| Note: Standard errors in parenthesis, ^*^ *p* < 0.05, ^**^ *p* < 0.01, ^***^ *p* < 0.001 (2-sided cluster-robust p-values), LCA estimated using STATA lclogit2 with membership. Four class model failed to converge. | | | |

## Appendix 8: Testing different linear specifications

To test for non-linearity in the daily bowel frequency, 10-year risk of needing a permanent stoma, and risk of short-term complication attributes, additional models were estimated. The first model specified all attributes as categorial. The second model specified all three attributes as linear. The third model tested whether adding a polynomial specification improved model fit, and the fourth model tested whether replacing attributes with a logarithmic transformation improved model fit. Where appropriate, the likelihood-ratio tests were conducted to assess model performance. Non-linear specifications in models 3 and 4 did not improve model fit based on AIC, BIC, and likelihood-ratio tests, and Model 2 had the lowest BIC/ AIC scores.

| **Variables** | **Model 1: MNL All Categorical model** | **Model 2: MNL with linear attributes** | **Model 3: MNL with linear and squared terms** | **Model 4: MNL with logarithmic attributes** |
| --- | --- | --- | --- | --- |
| Surgery A or B | 0.000 (.) | 0.000 (.) | 0.000 (.) | 0.000 (.) |
| Medication opt-out | 0.603^***^ (0.171) | 0.640^***^ (0.141) | 0.665^***^ (0.148) | 0.510^***^ (0.143) |
| **Improvement of Quality of life** | | | | |
| Mild | 0.000 (.) | 0.000 (.) | 0.000 (.) | 0.000 (.) |
| Moderate | 0.410^***^ (0.067) | 0.426^***^ (0.066) | 0.426^***^ (0.066) | 0.418^***^ (0.066) |
| High | 0.562^***^ (0.081) | 0.585^***^ (0.078) | 0.583^***^ (0.078) | 0.581^***^ (0.078) |
| **Daily bowel frequency** | | | | |
| 7 times | 0.000 (.) |  |  |  |
| 6 times | -0.005 (0.123) |  |  |  |
| 5 times | 0.301^**^ (0.093) |  |  |  |
| 4 times | 0.582^***^ (0.109) |  |  |  |
| 3 times | 0.717^***^ (0.117) |  |  |  |
| 2 times | 0.819^***^ (0.108) |  |  |  |
| Linear bowel frequency |  | -0.180^***^ (0.019) | -0.119 (0.099) |  |
| Linear bowel frequency^2 |  |  | -0.007 (0.011) |  |
| Logarithmic bowel frequency |  |  |  | -0.678^***^ (0.074) |
| **Bowel urgency** | | | | |
| Need to hurry to the toilet | 0.000 (.) | 0.000 (.) | 0.000 (.) | 0.000 (.) |
| Can delay for up to 15 mins | 0.915^***^ (0.093) | 0.943^***^ (0.091) | 0.944^***^ (0.092) | 0.944^***^ (0.091) |
| Can delay as long as needed | 1.293^***^ (0.096) | 1.306^***^ (0.095) | 1.307^***^ (0.095) | 1.304^***^ (0.095) |
| **Having a stoma** | | | | |
| No stoma | 0.000 (.) | 0.000 (.) | 0.000 (.) | 0.000 (.) |
| Temporary stoma | -0.244^***^ (0.067) | -0.260^***^ (0.066) | -0.261^***^ (0.066) | -0.265^***^ (0.066) |
| Temporary stoma # Male shift |  |  |  |  |
| Reversible stoma | -0.506^***^ (0.068) | -0.531^***^ (0.068) | -0.535^***^ (0.068) | -0.520^***^ (0.068) |
| Reversible stoma # Male shift |  |  |  |  |
| Permanent stoma | -1.488^***^ (0.138) | -1.430^***^ (0.125) | -1.436^***^ (0.126) | -1.399^***^ (0.122) |
| Permanent stoma # Male shift |  |  |  |  |
| **10-year risk of needing a permanent stoma** | | | | |
| No risk | 0.000 (.) |  |  |  |
| 5% | -0.043 (0.075) |  |  |  |
| 10% | -0.267^**^ (0.095) |  |  |  |
| 20% | -0.325^***^ (0.084) |  |  |  |
| Linear 10-year risk of stoma |  | -0.017^***^ (0.004) | -0.017^***^ (0.004) | -0.017^***^ (0.004) |
| **Risk of short-term complications** | | | | |
| 5% | 0.000 (.) |  |  |  |
| 10% | 0.052 (0.085) |  |  |  |
| 20% | -0.240^**^ (0.083) |  |  |  |
| 30% | -0.268^**^ (0.082) |  |  |  |
| Linear short-term complications |  | -0.014^***^ (0.003) | -0.014^***^ (0.003) |  |
| Logarithmic short-term complications |  |  |  | -0.198^***^ (0.038) |
| **Fertility** |  |  |  |  |
| Women:19%; Men:0% | 0.000 (.) | 0.000 (.) | 0.000 (.) | 0.000 (.) |
| Women:25%; Men:0% | -0.056 (0.086) | -0.062 (0.087) | -0.064 (0.087) | -0.060 (0.088) |
| Women:25%; Men:0% # Plan to have children |  |  |  |  |
| Women:40%; Men:0% | -0.201^*^ (0.079) | -0.173^*^ (0.079) | -0.180^*^ (0.078) | -0.151 (0.079) |
| Women:40%; Men:0% # Plan to have children |  |  |  |  |
| Women:43%; Men:15% | -0.259^***^ (0.075) | -0.239^**^ (0.073) | -0.243^***^ (0.073) | -0.231^**^ (0.073) |
| Women:43%; Men:15% # Plan to have children |  |  |  |  |
| Observations | 3150 | 3150 | 3150 | 3150 |
| Log-likelihood | -3035.66 | -3042.05 | -3041.86 | -3046.39 |
| BIC | 6272.71 | **6212.26** | 6221.03 | 6220.94 |
| AIC | 6115.33 | **6112.10** | 6113.73 | 6120.79 |
| Likelihood-ratio test chi2 (p-value) | - | 12.77 (0.1199) (model 2 nested within model 1) | 0.38 (0.5398) (model 2 nested within model 3) | - |
| Standard errors in parentheses; ^*^ *p* < 0.05, ^**^ *p* < 0.01, ^***^ *p* < 0.001 (Robust 2-sided) | | | | |

## Appendix 9: Selection of draws

The table below shows the parameter estimates of three mixed logit models, estimated using 1000, 2000, and 5000 MLHS draws. The last two columns compare the stability of the estimated parameters compared with the parameters of the model with 5000 MLHS draws. The percentage differences are mostly stable across the models. However, there are some substantial percentage differences in the standard deviations for several attribute levels, but in most cases, the SD parameters were not statistically significant.

| **Variables** | **Estimate_MLHS_1000** | **Estimate_MLHS_2000** | **Estimate_MLHS_5000** | **5000-1000_%_ difference** | **5000-2000_%_ difference** |
| --- | --- | --- | --- | --- | --- |
| Medication Mean | -0.571 | -0.641* | -0.565* | 1.00 | 13.43 |
| Medication SD | 4.047*** | 3.975*** | 4.016*** | 0.77 | -1.03 |
| QoL Moderate Mean | 0.541*** | 0.536*** | 0.548*** | -1.27 | -2.07 |
| QoL Moderate SD | -0.327 | 0.223 | -0.276 | 18.23 | -180.52 |
| QoL High Mean | 0.868*** | 0.867*** | 0.876*** | -0.98 | -1.08 |
| QoL High SD | 0.591* | -0.576** | 0.614** | -3.67 | -193.91 |
| Bowel Freq Mean | -0.291*** | -0.288*** | -0.291*** | 0.17 | -0.95 |
| Bowel Freq SD | -0.228*** | 0.202*** | 0.202*** | -212.87 | 0.08 |
| Delay for 15 mins Mean | 1.395*** | 1.319*** | 1.358*** | 2.69 | -2.87 |
| Delay for 15 mins SD | -0.076 | 0.042 | -0.022 | 251.38 | -293.06 |
| Delay as long as needed Mean | 1.936*** | 1.847*** | 1.887*** | 2.59 | -2.11 |
| Delay as long as needed SD | -0.938*** | -0.912*** | -0.934*** | 0.44 | -2.28 |
| Temporary stoma Mean | -0.313* | -0.270* | -0.284* | 9.97 | -4.96 |
| Temporary stoma SD | -0.399* | -0.361 | 0.311 | -228.39 | -215.92 |
| Temporary stoma Shift Men | -0.174 | -0.199 | -0.194 | -9.97 | 2.75 |
| Reversible stoma Mean | -0.569*** | -0.544*** | -0.556*** | 2.45 | -2.14 |
| Reversible stoma SD | -0.488** | 0.392* | 0.467** | -204.48 | -16.00 |
| Reversible stoma Shift Men | -0.563** | -0.555** | -0.564** | -0.16 | -1.48 |
| Permanent stoma Mean | -2.210*** | -2.105*** | -2.153*** | 2.66 | -2.21 |
| Permanent stoma SD | 1.530*** | -1.472*** | -1.521*** | -200.60 | -3.19 |
| Permanent stoma Shift Men | -0.888** | -0.905** | -0.890* | -0.26 | 1.73 |
| 10-year stoma risk Mean | -0.039*** | -0.037*** | -0.038*** | 2.66 | -1.88 |
| 10-year stoma risk SD | 0.033* | 0.021 | 0.031* | 8.41 | -29.79 |
| Short term risk Mean | -0.027*** | -0.026*** | -0.026*** | 2.87 | -0.79 |
| Short term risk SD | -0.027** | -0.024* | -0.024** | 13.07 | -0.53 |
| Fertility: Women:25%; Men:0% Mean | 0.05 | 0.037 | 0.048 | 4.09 | -23.52 |
| Fertility: Women:25%; Men:0% SD | 0.378** | -0.166 | 0.300 | 25.98 | -155.44 |
| Fertility: Women:25%; Men:0% Shift Family planning | -0.584* | -0.548* | -0.575* | 1.65 | -4.57 |
| Fertility: Women:40%; Men:0% Mean | 0.013 | 0.000 | 0.01 | 22.91 | -103.45 |
| Fertility: Women:40%; Men:0% SD | -0.085 | -0.048 | -0.024 | 254.19 | 99.33 |
| Fertility: Women:40%; Men:0% Shift Family planning | -0.804*** | -0.771*** | -0.801** | 0.37 | -3.73 |
| Fertility: Women:43%; Men:15% Mean | -0.211 | -0.204 | -0.201 | 5.19 | 1.52 |
| Fertility: Women:43%; Men:15% SD | 0.418* | 0.317 | -0.374 | -211.69 | -184.88 |
| Fertility: Women:43%; Men:15% Shift Family planning | -0.721** | -0.706** | -0.736** | -2.04 | -4.04 |
| Observations | 3150 | 3150 | 3150 |  |  |
| Log-likelihood | -2252.83 | -2253.89 | -2253.28 |  |  |
| AIC | 4573.65 | 4575.77 | 4574.55 |  |  |
| BIC | 4779.53 | 4781.65 | 4780.43 |  |  |
| Estimation time | 14 mins | 28 mins | 7 hours 7 mins |  |  |
| Notes: * p < 0.05, ** p < 0.01, *** p < 0.001 (2-sided Robust p-values). Mixed logit models were estimated using the BGW algorithm (11) with Modified Latin Hypercube Sampling draws (12). | | | | | |

## Appending 10: Internal Validity

Overall, the data was of high internal validity, and the table below provides the results of these internal validity checks. The risk comprehension test (see Figure 1 below) was included to check whether participants understood probabilities, where one alternative was clearly superior. Over 97% passed the risk comprehension test, indicating a high level of comprehension. Similarly, 94.6% passed the “check your understanding” quiz question (see Figure 2), a multiple-choice question used to verify respondents' comprehension of the attribute details provided on the previous screen. Participants who answered incorrectly were immediately provided with an explanation and correction. The survey was hosted on Qualtrics, which has built-in tests to detect bots or duplicate entries. The results show that over 98% of the respondents that completed the survey can be classed as genuine. Only three chose the same surgical alternative (either Treatment A or Treatment B) for all nine DCE tasks (straight-lined). These respondents were not excluded from the final analysis, as the number is very small, these responses are less likely to bias the overall model results. The 43 respondents who consistently chose the medication opt-out were considered to have a genuine preference rather than a form of poor responding. In the comments section, some of the respondents explained why they always chose the medication opt-out. These include a high aversion to the infertility risk associated with surgery and the general fear or anxiety surrounding the surgical procedure itself. The majority of participants (89%) reported that they understood the questions about making choices between different treatment options. Although most understood the questions, a sizable proportion of participants found the actual trade-offs difficult (38%) and wanted more information (34%).

|  | No. | % |
| --- | --- | --- |
| **Quality assurance questions** | | |
| Failed risk comprehension test | | |
| No | 342 | 97.70% |
| Yes | 8 | 2.30% |
| Failed understanding attribute information test | | |
| No | 331 | 94.6% |
| Yes | 19 | 5.4% |
| Failed Qualtrics bot detection test | | |
| No | 346 | 98.9% |
| Yes | 3 | 0.9% |
| Missing | 1 | 0.3% |
| Failed Qualtrics Duplicate test | | |
| No | 348 | 99.4% |
| Missing | 2 | 0.6% |
| Choose surgery A or surgery B all the way (straight-lined)^a^ | | |
| No | 347 | 99.1% |
| Yes | 3 | 0.9% |
| **Feedback questions** | | |
| I understood the questions about making choices between different treatment options | | |
| Agree/Strongly Agree | 313 | 89.4% |
| Uncertain | 5 | 1.4% |
| Disagree/Strongly Disagree | 32 | 9.2% |
| When choosing between different treatment options I needed more information | | |
| Agree/Strongly Agree | 120 | 34.3% |
| Uncertain | 40 | 11.4% |
| Disagree/Strongly Disagree | 190 | 54.3% |
| I found making a choice between different treatment options difficult | | |
| Agree/Strongly Agree | 132 | 37.7% |
| Uncertain | 37 | 10.6% |
| Disagree/Strongly Disagree | 181 | 51.7% |
| ^a^This excludes the 43 who choose the medication opt-out throughout, we considered this a genuine preference as opposed to straight-lining. | | |

**Figure 1: Risk comprehension test**

**Figure 2: Check your understanding quiz task**

## Appendix 11: Conditional probabilities

**Figure: Table 3 results reproduced visually**

**Figure: Treatment Uptake by clusters**

## References

1.	Allison M, Lindsay J, Gould D, et al. Surgery in young adults with inflammatory bowel disease: a narrative account. International journal of nursing studies. 2013; 50: 1566-75.

2.	Coates E, Wickramasekera N, Barr A, et al. Patient preferences and current practice for adults with steroid-resistant ulcerative colitis: POPSTER mixed-methods study. Health Technology Assessment (Winchester, England). 2022; 26: 1.

3.	Fourie S, Jackson D, Aveyard H. Living with inflammatory bowel disease: a review of qualitative research studies. International Journal of Nursing Studies. 2018; 87: 149-56.

4.	Rapport F, Clement C, Seagrove AC, et al. Patient views about the impact of ulcerative colitis and its management with drug treatment and surgery: a nested qualitative study within the CONSTRUCT trial. BMC gastroenterology. 2019; 19: 166.

5.	Bewtra M, Kilambi V, Fairchild AO, et al. Patient preferences for surgical versus medical therapy for ulcerative colitis. Inflammatory bowel diseases. 2014; 20: 103-14.

6.	Schoefs E, Vermeire S, Ferrante M, et al. What are the unmet needs and most relevant treatment outcomes according to patients with inflammatory bowel disease? A qualitative patient preference study. Journal of Crohn's and Colitis. 2023; 17: 379-88.

7.	Lai C, Sceats L, Qiu W, et al. Patient decision‐making in severe inflammatory bowel disease: the need for improved communication of treatment options and preferences. Colorectal Disease. 2019; 21: 1406-14.

8.	Siegel CA, Schwartz LM, Woloshin S, et al. When should ulcerative colitis patients undergo colectomy for dysplasia? Mismatch between patient preferences and physician recommendations. Inflammatory bowel diseases. 2010; 16: 1658-62.

9.	Lee M, Folan A, Baker D, et al. A survey of patient informational preferences when choosing between medical and surgical therapy for ulcerative colitis: a sub‐study from the DISCUSS project. Colorectal Disease. 2023.

10.	Yang ML, Brar MS, Kennedy E, et al. Three‐stage versus modified two‐stage surgery for ulcerative colitis: a patient‐centred treatment trade‐off study. Colorectal Disease. 2024; 26: 1184-90.

11.	Bunch DS, Gay DM, Welsch RE. Algorithm 717: Subroutines for maximum likelihood and quasi-likelihood estimation of parameters in nonlinear regression models. ACM Transactions on Mathematical Software (TOMS). 1993; 19: 109-30.

12.	Hess S, Train KE, Polak JW. On the use of a Modified Latin Hypercube Sampling (MLHS) method in the estimation of a Mixed Logit model for vehicle choice. Transportation Research Part B: Methodological. 2006; 40: 147-63.

13.	Ormerod CR. The IBD Control Questionnaire: The Development and Psychometric Validation of a Questionnaire for Measuring Inflammatory Bowel Disease Control from the Patient's Perspective. The University of Liverpool (United Kingdom), 2019.

14.	Walker JL, Wang Y, Thorhauge M, et al. D-efficient or deficient? A robustness analysis of stated choice experimental designs. Theory and Decision. 2018; 84: 215-38.
